# Functional analysis of *SLC39A8* mutations and their implications for manganese deficiency and mitochondrial disorders

**DOI:** 10.1038/s41598-018-21464-0

**Published:** 2018-02-16

**Authors:** Eun-Kyung Choi, Trang-Tiffany Nguyen, Neil Gupta, Shigeki Iwase, Young Ah Seo

**Affiliations:** 1 Department of Nutritional Sciences, University of Michigan School of Public Health, Ann Arbor, MI 48109 USA; 2 Department of Human Genetics, University of Michigan, Ann Arbor, MI 48109 USA

## Abstract

*SLC39A8* encodes ZIP8, a divalent metal ion transporter. Mutations in the *SLC39A8* gene are associated with congenital disorder of glycosylation type II and Leigh syndrome. Notably, affected patients with both disorders exhibited severe manganese (Mn) deficiency. The cellular function of human *SLC39A8* (*hSLC39A8*) and the mechanisms by which mutations in this protein lead to human diseases are unclear. Herein, we show that *hSLC39A8* mediates ^54^Mn uptake by the cells, and its expression is regulated by Mn. While expression of wild-type *hSLC39A8* increased ^54^Mn uptake activity, disease-associated mutations abrogated the ability of the transporter to mediate Mn uptake into the cells, thereby providing a causal link to severe Mn deficiency. All mutants failed to localize on the cell surface and were retained within the endoplasmic reticulum. Interestingly, expression of *hSLC39A8* mutants of both CDG type II and Leigh syndrome reduced mitochondrial ^54^Mn levels and activity of Mn-dependent mitochondrial superoxide dismutase MnSOD, and in turn increased oxidative stress. The expression of wild-type *hSLC39A8*, but not the disease-associated mutants, promoted mitochondrial functions. Moreover, loss of function analyses further corroborate *hSLC39A8*’s critical role in mediating Mn uptake and mitochondrial function. Our results provide a potential pathogenic mechanism of diseases that are associated with *hSLC39A8* mutations.

## Introduction

Manganese (Mn) is an essential nutrient that acts as a cofactor for a variety of enzymes involved in numerous cellular physiological processes^1^. However, when accumulated at high levels, Mn can be a potent toxicant to cells, as it increases oxidative stress, impairs mitochondrial function, and promotes cell death^2,3^. The essential yet toxic nature of Mn necessitates precise homeostatic mechanisms to maintain appropriate levels of intracellular Mn. Thus, cells require efficient transport mechanisms for the uptake, intracellular distribution, and efflux of metal ions. Compared to other essential metals, however, the transport and homeostatic processes of Mn are not well-defined^1^.

The gene *SLC39A8* (ZIP8, MIM #608732) is a member of the Zrt- and Irt-like protein (ZIP) family of metal transporters^4^. These family proteins were initially found to transport zinc into the cytoplasm, either across the plasma membrane or out of intracellular organelles^4^. In addition to zinc, *in vitro* studies have shown that *SLC39A8* can transport Mn^5^, iron^6^, and cadmium^5^, with a higher affinity with Mn than zinc in mammalian cells^5^. Both *SLC39A8* global and liver-specific knockout mice displayed decreased tissue Mn levels, with no differences in zinc and iron levels^7^. Thus, the specificity of *SLC39A8* in metal transport remains undefined.

The recent identification of genetic mutations in *SLC39A8* has highlighted the consequences of dysregulated Mn homeostasis for human health. In 2015, two studies simultaneously identified *SLC39A8* mutations in the congenital disorder of glycosylation type II (CDG type II; OMIM #616721)^8,9^, a severe multisystem developmental disorder characterized by delayed psychomotor development^8,9^. The major molecular feature of CDG type II is the defect in processing protein-bound glycans either late in the endoplasmic reticulum (ER) or in the Golgi compartments^10^. Based on the reduced glycosylation of transferrin in patient cells, it has been speculated that *SLC39A8* is essential for supplying Mn to the Mn-dependent β-1,4-galactosyltransferase, the central enzyme mediating protein glycosylation^9^.

In 2016, another *SLC39A8* mutation was identified in Leigh syndrome, an early-onset progressive neurodegenerative disorder characterized by defects in mitochondrial energy production (OMIM #256000)^11^. The association of Leigh syndrome with the *SLC39A8* mutation was somewhat unexpected. While *SLC39A8* is primarily found in the plasma membrane^12^, Leigh syndrome is usually caused by mutations in mitochondrial components. Notably, affected patients with both disorders exhibited severe Mn deficiency in the blood^8,9,11^. These observations raised the possibility that human *SLC39A8* (*hSLC39A8*) is a Mn transporter and the disease-associated *hSLC39A8* mutations dysregulate Mn homeostasis. However, this hypothesis has not been tested directly.

While the importance of *hSLC39A8* in multiple disease processes is increasingly recognized^8,9,11^, little is known regarding the underlying mechanisms of *hSLC39A8* in cellular Mn homeostasis, how these mutations lead to human disease^8,9,11^. This study aimed to determine the function of *hSLC39A8* in cellular Mn uptake and how disease-associated mutations affect cellular Mn homeostasis, and to explore the functional consequences of these mutations. Herein, we report that the expression of wild-type *hSLC39A8* (*hSLC39A8*-WT) significantly increased ^54^Mn uptake activity and intracellular Mn levels. However, disease-associated *hSLC39A8* mutations completely abrogated the ^54^Mn uptake activity of *hSLC39A8*, thereby providing a causal link to severe Mn deficiency. Furthermore, the expression of *hSLC39A8* mutants significantly reduced mitochondrial ^54^Mn levels and the activity of the Mn-dependent mitochondrial superoxide dismutase MnSOD. Importantly, the expression of *hSLC39A8*-WT potentiates mitochondrial functions, while *hSLC39A8* mutants are less capable of doing so and, rather, increase oxidative stress. Both loss of function and gain of function analysis suggest that *hSLC39A8* plays a critical role in Mn uptake and mitochondrial function, which likely contribute to the pathogenesis of diseases that are associated with *hSLC39A8* mutations.

## Results

### *hSLC39A8* is a Mn-specific transporter

**Figure 1:**
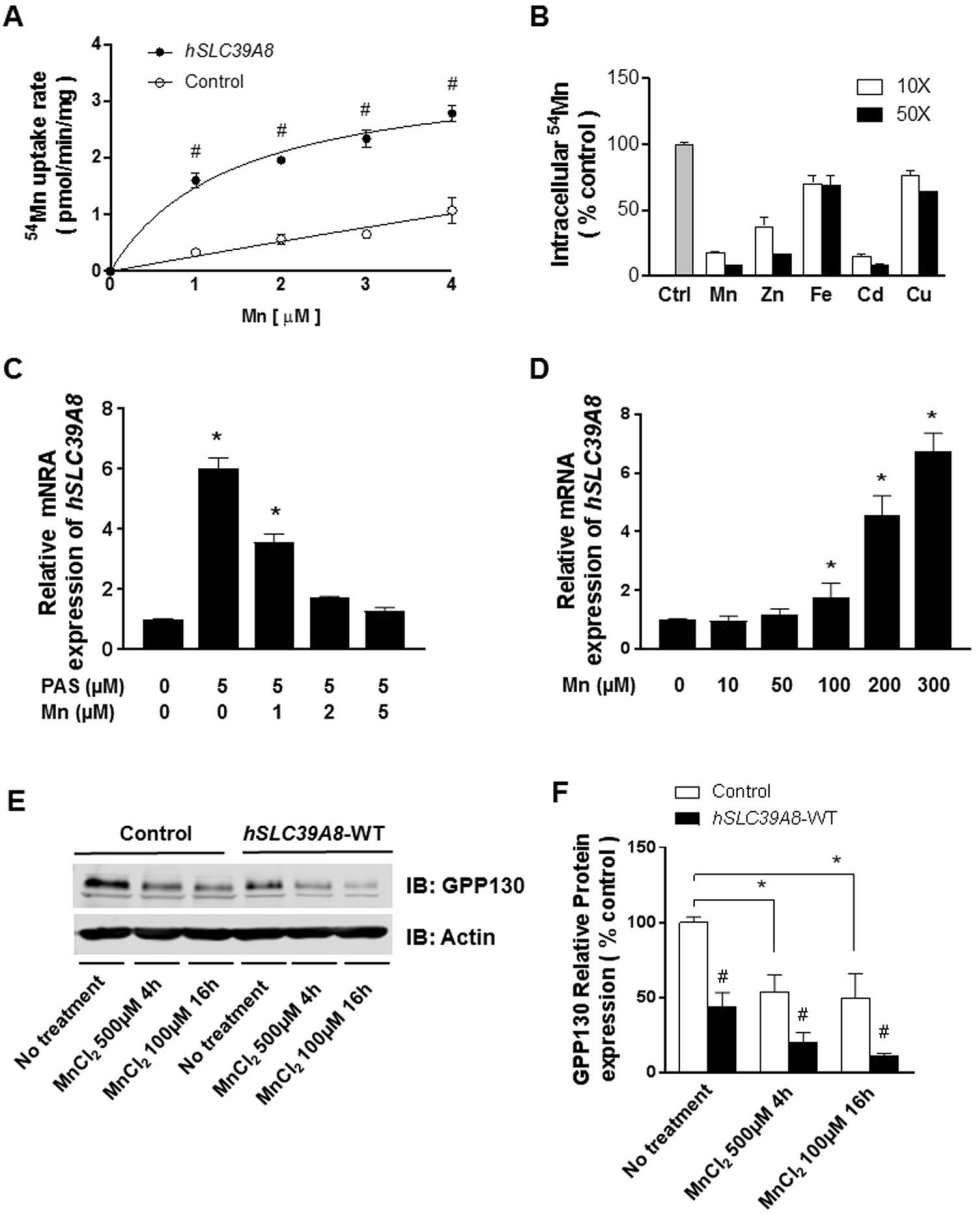
*hSLC39A8* is a Mn-specific transporter and its expression is regulated by Mn. (**A**) Concentration-dependent Mn uptake (pmol/min/mg protein) by HeLa cells transfected to express *hSLC39A8* (closed circles) or empty vector (open circles). Data represent means ± SEM (n = 2 samples/group). ^#^*P* < 0.05 vs. control; Student’s *t*-test. (**B**) Competition assays with HeLa cells transfected to express *hSLC39A8*. ^54^Mn uptake was measured in the presence of 10- or 50-fold molar excess of the metals indicated. Ctrl refers to control with no metals added. (**C**) *hSLC39A8* expression under various Mn concentrations, with or without the Mn chelator PAS, and (**D**) under increasing Mn concentrations for 16 h. Mean relative transcript levels are detected by qPCR. Data represent means ± SEM (n = 3 samples/group). ^#^*P* < 0.05 vs. control: Student’s *t*-test. (**E**) Representative immunoblot of GPP130 in total cell lysates isolated from empty vector- (control), or *hSLC39A8*-WT expressing cells incubated with 500 µM MnCl_2_ for 4 h or 100 µM for 16 h. Equal loading was verified by immunoblotting with actin antibody. Full-length blots are presented in Supplementary Figure 3. (**F**) Quantification of GPP130 relative protein after normalization with actin. Results are means ± SEM (n = 3 samples/group) of three independent experiments. ^#^*P* < 0.05 vs. control at the same concentration, *P < 0.05 vs. control in the different groups; Student’s *t*-test.

To directly test whether *hSLC39A8* mediates Mn uptake, we measured ^54^Mn uptake into the cells. As shown in Fig. 1A, *hSLC39A8*-transfected cells strongly stimulated the uptake of ^54^Mn compared to empty vector (control)-transfected cells. *hSLC39A8*-dependent ^54^Mn uptake was concentration-dependent and saturable, with an apparent Km of ~1.44 ± 0.39 μM (Fig. 1A). To assess the substrate specificity of *hSLC39A8*, competition assays were performed with cells expressing *hSLC39A8* in the presence of excess non-radioactive metal cations. Figure 1B shows the degree to which different metal ions compete with Mn uptake. The application of a 10- or 50-fold excess of non-radioactive Mn and Cd caused a marked decrease in ^54^Mn uptake by the cells, with the order of the inhibitory effect expressed as Mn = Cd > Zn > Fe = Cu. These results indicate that *hSLC39A8* mediates Mn uptake into the cells with a very high affinity, and cadmium has the strongest inhibitory effect on Mn uptake. These observations are consistent with previous reports on *SLC39A8*^5,7^ as well as other mammalian SLC39 transporters^13,14^.

We then investigated whether Mn regulates the expression of *hSLC39A8*. The HeLa cells were treated with a Mn chelator, para-aminosalicylic acid (PAS), with a range of Mn concentrations. The mRNA levels of endogenous *hSLC39A8* were determined by quantitative PCR (qPCR). As shown in Fig. 1C, *hSLC39A8* expression was elevated ~6-fold by Mn-limiting conditions compared to that in the basal medium (Fig. 1C). Moreover, various Mn concentrations were tested until the *hSLC39A8* mRNA level reached the plateau. Figure 1D shows that *hSLC39A8* mRNA levels were significantly upregulated by ~1.7-fold (*P* = 0.02), ~4.6-fold (*P* < 0.001), and ~6.8-fold (*P* < 0.0001) with Mn treatments of 100, 200, and 300 µM, respectively (Fig. 1D). The increased expression of endogenous *hSLC39A8* by Mn implies the existence of a homeostatic mechanism, by which *hSLC39A8* maintains optimal Mn levels within cells.

### Mn sensor GPP130 validates the Mn uptake activity of *hSLC39A8*

To further test whether *hSLC39A8* functions as a Mn uptake transporter, we used the Golgi protein GPP130, which is known to be a specific Golgi Mn sensor^15^. Previous studies have shown that a specific increase in intra-Golgi Mn led to GPP130 trafficking from the Golgi to late endosomes and lysosomes, where GPP130 was degraded^15^. Thus, we hypothesized that if *hSLC39A8* is a Mn-specific uptake protein, it will uptake Mn from the cell exterior into the cytoplasm and provide the metal to the Golgi, inducing GPP130 degradation. The stability of GPP130 was assessed with and without MnCl_2_ treatment by immunoblot in cells expressing *hSLC39A8* (Fig. 1E). Consistent with previous studies^16,17^, the level of GPP130 was significantly reduced when control cells were treated with Mn (Fig. 1E,F). Importantly, this Mn-induced degradation was significantly reduced in cells expressing *hSLC39A8* compared to control cells treated with Mn. Quantification indicated that 79% of GPP130 was lost in cells expressing *hSLC39A8*-WT after 4 h Mn treatment, while only a 46% decrease was seen in control cells (Fig. 1F). To test if GPP130 is indeed degraded in the cells expressing *hSLC39A8*, we pretreated cells with the lysosomal inhibitor NH_4_Cl. The pretreatment blocked the Mn-induced reduction of GPP130 levels, indicating that GPP130 is degraded by lysosome upon *SLC39A8* expression (Supplementary Fig. 1). Taken together, these data validate that *hSLC39A8* is a key Mn transporter in mammalian cells.

### Disease-associated mutations impair Mn uptake activity of *hSLC39A8*

**Figure 2:**
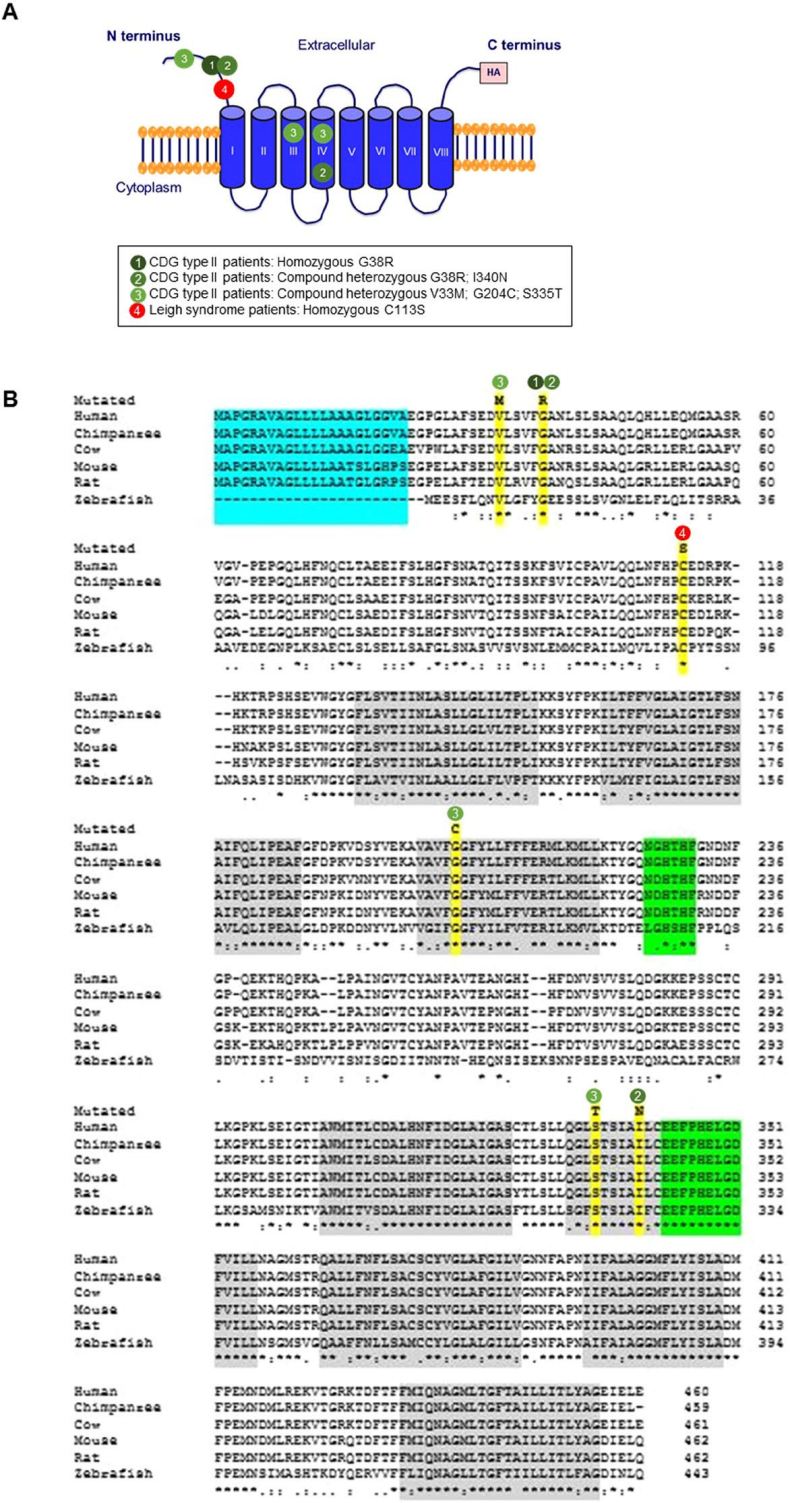
Mutations in *hSLC39A8* are associated with severe Mn deficiency, congenital disorder of glycosylation type II, and/or Leigh syndrome. (**A**) A model of predicted topology of human *SLC39A8* is shown with eight transmembrane domains, extracellular NH_2_ or COOH terminus, and the COOH terminal HA tag used in these experiments. Four mutations identified in patients with Mn deficiency were examined in this study and the corresponding locations of these mutations are indicated. (**B**) Evolutionary alignment of *SLC39A8* amino acid sequence showing strict conservation of Mn deficiency-associated *hSLC39A8* mutations. Protein sequences for *SLC39A8* were aligned using ClustalW2. Residues identical to the human *SLC39A8* sequence are marked with an asterisk (*). Conservation between amino acids of strongly and weakly similar properties is indicated by a colon (:) and a period (.), respectively. Putative protein domains are predicted using MEMSATSVM and include a signaling peptide in turquoise (position 1–22), a histidine-rich region in green (position 226–231), and the metalloprotease motif in green (position 343–351). Disease-associated mutations are highlighted in yellow (V33M, G38R, C113S, G204C, S335T, I340N). The protein sequences used to generate this alignment include NP_001128618.1 (human), JAA33667.1 (chimpanzee), NP_001192559.1 (cow), NP_001128622.1 (mouse), AAH89844.1 (rat), and XP_009305480.1 (zebrafish).

Mutations in *hSLC39A8* were identified to cause severe Mn deficiency in patients with CDG type II and Leigh syndrome^8,9,11^. To determine the impact of these mutations on Mn transport, we constructed an expression vector encoding *hSLC39A8* with a C-terminal HA epitope tag and introduced four disease-associated mutations into *hSLC39A8*. Four mutations included two missense and two compound missense mutations of *SLC39A8* that are found in CDG type II or Leigh syndrome patients. The mutations and their localization are summarized in Fig. 2A. The first mutation associated with CDG type II patients (*hSLC39A8*-M1, G38R) was a homozygous c.112 G > C substitution. The second mutation associated with CDG type II patients (*hSLC39A8*-M2, G38R; I340N) was a compound heterozygous for c.112 G > C and c.1019 T > A. The third mutation associated with CDG type II patients (*hSLC39A8*-M3, V33M; G204C; S335T) was a compound heterozygous for c.97 G > A, c.610 G > T, and c.1004 G > C. The fourth mutation associated with Leigh syndrome (*hSLC39A8*-M4, C113S) was a homozygous c.338 G > C substitution. All four mutants are missense changes that affect highly conserved amino acid residues (Fig. 2B).

**Figure 3:**
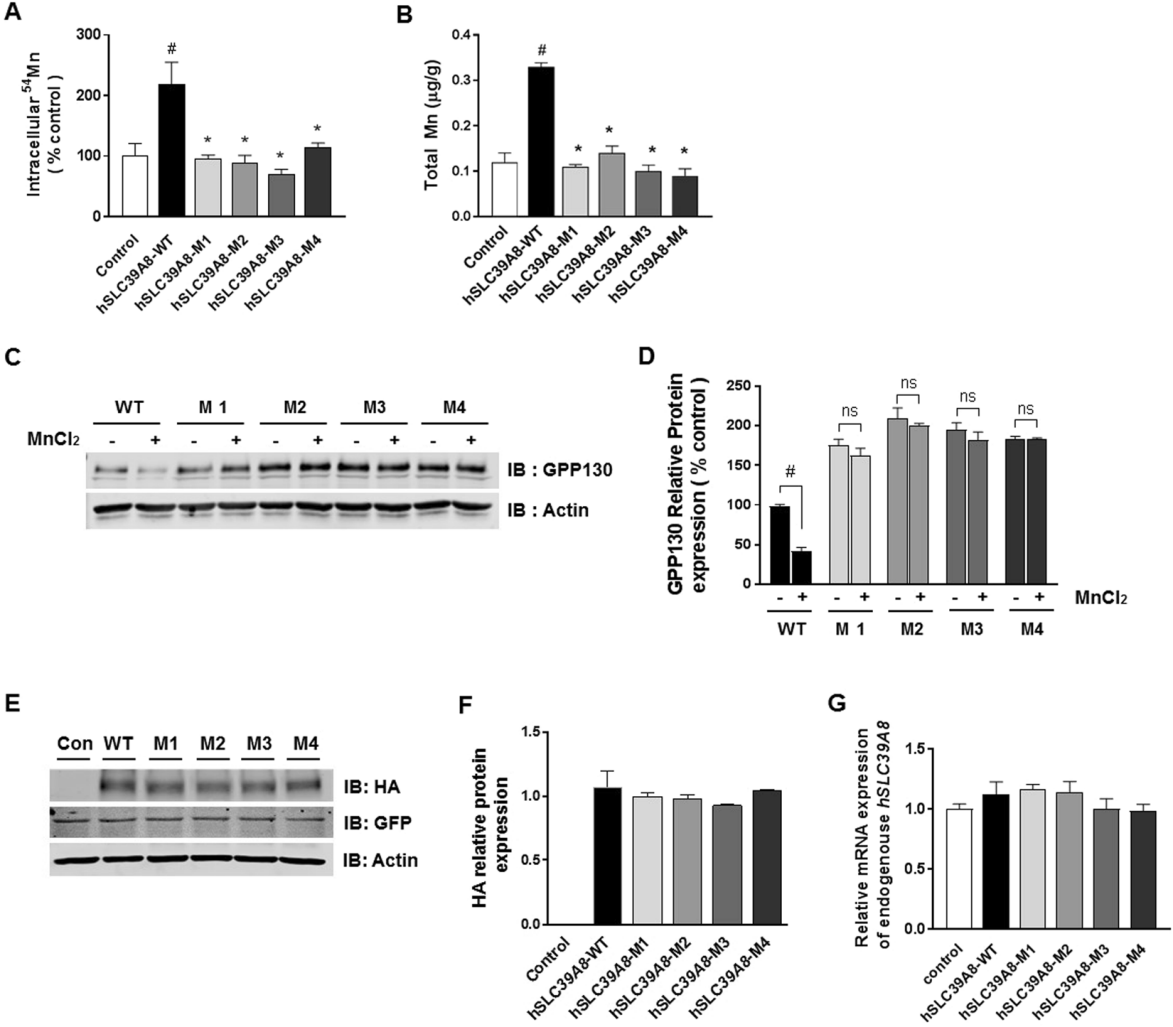
Disease-associated *hSLC39A8* mutants reduce ^54^Mn uptake and intracellular Mn levels. (**A**) HeLa cells were transfected with *hSLC39A8*-WT or *hSLC39A8* mutants for 48 h and then incubated at 37 °C for 30 min in the presence of 1 µM of ^54^Mn. Cells were chilled on ice and washed with ice-cold PBS containing 1 mM EDTA, and cell-associated radioactivity was determined by gamma counting and normalized to control vector-expressing cells. Data represent means ± SEM (n = 3 samples/group). (**B**) HeLa cells were transfected with *hSLC39A8*-WT or *hSLC39A8* mutants for 48 h. Intracellular metal levels, including Mn, were measured by ICP-MS. Data represent means ± SEM (n = 3 samples/group). (**C**) Representative immunoblot of GPP130 in total cell lysates isolated from *hSLC39A8*-WT expressing cells and its mutants incubated with MnCl_2_ 500 µM for 4 h. Equal loading was verified by immunoblotting with actin antibody. Full-length blots are presented in Supplementary Figure 4. (**D**) Quantification of GPP130 relative protein after normalization with actin. (**E**) Representative immunoblot of *hSLC39A8*-WT-HA and its mutants in total cell lysates isolated from cells transfected with empty vector (control), *hSLC39A8*-WT-HA, or its mutant (*hSLC39A8*-M1-HA, -M2-HA, -M3-HA, and -M4-HA). GFP is included as a control for transfection efficiency. Equal loading was verified by immunoblotting with actin antibody and the positions of molecular weight markers are shown. Full-length blots are presented in Supplementary Figure 5. (**F**) Quantification of *hSLC39A8*-HA relative protein after normalization with actin. Results are means ± SEM of three independent experiments. (**G**) The endogenous expression of *SLC39A8* mRNA in cells expressing *SLC39A8*-WT or its mutants. Mean relative transcript levels are detected by qPCR. Data represent means ± SEM (n = 3 samples/group). ^#^*P* < 0.05 between control vector vs. *hSLC39A8*-WT or *P < 0.05 between *hSLC39A8*-WT vs. its mutants; Student’s *t*-test.

To probe the impact of these mutations on Mn transport activity, HeLa cells were transfected with the control, *hSLC39A8*-WT or *hSLC39A8*-mutants (*hSLC39A8*-M1, -M2, -M3, or -M4) and then assayed for ^54^Mn uptake activity. The expression of *hSLC39A8*-WT resulted in a ~2.2-fold increase (*P* = 0.008) in ^54^Mn uptake activity compared to the control cells (Fig. 3A). In contrast, all four mutants completely abrogated the increase in ^54^Mn uptake observed in cells expressing *hSLC39A8*-WT (*P* < 0.05). Moreover, the expression of *hSLC39A8*-WT significantly increased intracellular Mn levels (*P* < 0.05), whereas the expression of *hSLC39A8*-mutants did not (Fig. 3B). Despite the known ability of *SLC39A8* to transport zinc, iron, and cadmium, no differences were observed in intracellular zinc, iron, and copper levels in cells expressing *hSLC39A8*-WT or its mutants (Supplementary Fig. 2). Note that the intracellular cadmium levels were below the detection limit in cells expressing *hSLC39A8*-WT or mutants.

To further test the effect of *hSLC39A8* mutations on Mn transport activity, we again used GPP130. We hypothesized that if *hSLC39A8* mutations abrogate Mn uptake activity, they will inhibit GPP130 degradation. Consistent with our data in Fig. 1, the level of GPP130 was significantly reduced in cells expressing *hSLC39A8* treated with Mn (Fig. 3C,D). Importantly, expression of the disease-associated *hSLC39A8* mutants blocked the Mn-induced loss of GPP130 (Fig. 3C,D). Taken together, these data clearly indicate that the disease-associated mutations inhibit the Mn transporting activity of *hSLC39A8*. No difference was detected in protein levels between *hSLC39A8*-WT and mutations at the predicted molecular mass (~50 kDa) (Fig. 3E,F). No difference was detected in the endogenous expression of *SLC39A8* in cells expressing *hSLC39A8* and its mutants (Fig. 3G), confirming that Mn deficiency by *hSLC39A8* mutations was not affected by the endogenous expression of *hSLC39A8* in HeLa cells. These results, combined with the severe Mn deficiency observed in both CDG type II and Leigh syndrome^8,9,11^, demonstrate loss-of-function mechanisms underlying CDG type II and Leigh syndrome that are associated with *hSLC39A8* mutations.

### Disease-associated *hSLC39A8* mutants are mislocalized

**Figure 4:**
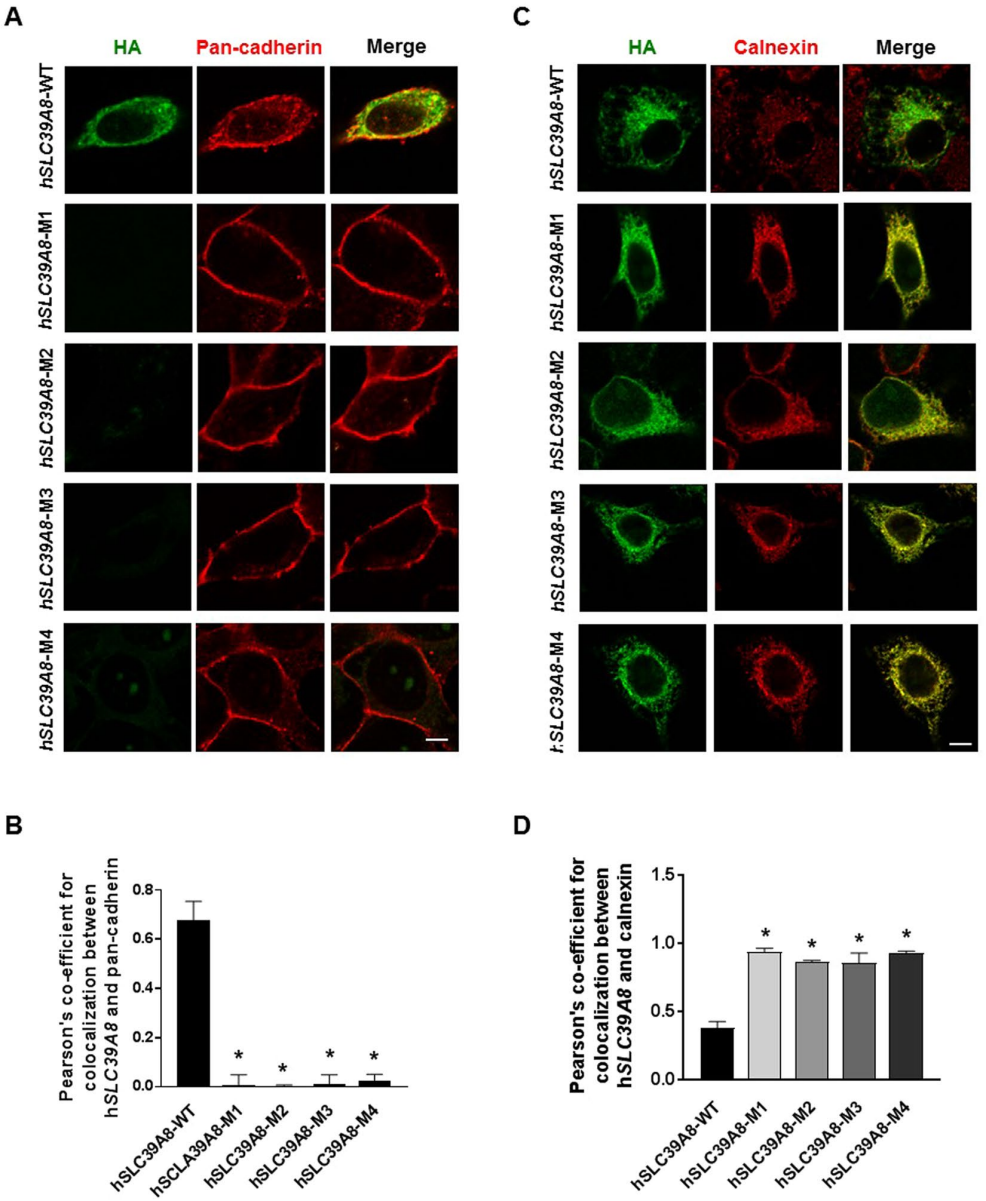
Disease-associated *hSLC39A8* mutants are mislocalized. (**A**) Representative confocal images of HeLa cells expressing the *hSLC39A8* and its mutants in non-permeabilized cells are shown. HeLa cells transfected with *hSLC39A8*-HA and other mutants were fixed and incubated without Triton X-100. To detect *hSLC39A8*-HA, the cells were incubated with Alexa 488-conjugated anti-mouse HA antibody (green). To detect the cell surface, the cells were coincubated with anti-pan cadherin antibody followed by Alexa Fluor 568-conjugated secondary antibody (red). (**B**) Quantification of the Pearson’s coefficient for colocalization between HA and calnexin from (**A**). (**C**) Representative confocal images of HeLa cells expressing the *hSLC39A8* and its mutants in permeabilized cells are shown. To detect *hSLC39A8* mutant, the cells were incubated with Alexa 488-conjugated anti-mouse HA antibody (green). To detect the ER, the cells were coincubated with anti-calnexin antibody followed by Alexa Fluor 568-conjugated secondary antibody (red). Scale bar = 10 µm. (**D**) Quantification of the Pearson’s coefficient for colocalization between HA and calnexin from (**C**). Results are means ± SEM of three independent experiments. *P < 0.05 between *hSLC39A8*-WT vs. other mutants; Student’s *t*-test. The value of PC can range from 1 to −1, with 1 standing for complete positive correlation and −1 for a negative correlation, with zero standing for no correlation.

The impaired Mn uptake activity observed in the pathogenic mutants may be caused by defects in localization and/or protein folding. To assess the effects of mutations on protein localization, we first examined the localization of HA-tagged *hSLC39A8*-WT. HeLa cells expressing *hSLC39A8*-WT were analyzed by confocal microscopy with or without the detergent Triton X-100, which permeabilizes the plasma membrane. In non-permeabilized cells, surface labeling with anti-HA antibody was strong for wild-type *hSLC39A8*-HA (Fig. 4A,B). Surface staining for all mutants was substantially weaker or undetectable. In permeabilized cells, *hSLC39A8*-WT was predominantly detected at the plasma membrane and throughout the cytosol with intracellular vesicle staining (Fig. 4C,D). In contrast, all mutant proteins showed clear staining of the nuclear envelope and a reticulated pattern of fluorescence extending from the nucleus into the cytoplasm, suggesting retention of the mutants within the ER. Our measurement of Pearson’s correlation coefficient^18,19^ showed statistically significant increases in HA−calnexin colocalization signals within mutant-expressing cells compared to WT-expressing cells (*P* < 0.0001, Fig. 4D). Taken together, our data indicate that *hSLC39A8*-WT is primarily localized on the cell surface, whereas the disease-associated mutants are retained in the ER. The failure of localization at the plasma membrane likely explains the inability of *hSLC39A8*-mutants to transport Mn into cells.

### The expression of *hSLC39A8*, but not its disease-associated mutants, led to increased mitochondrial Mn levels and MnSOD activity

**Figure 5:**
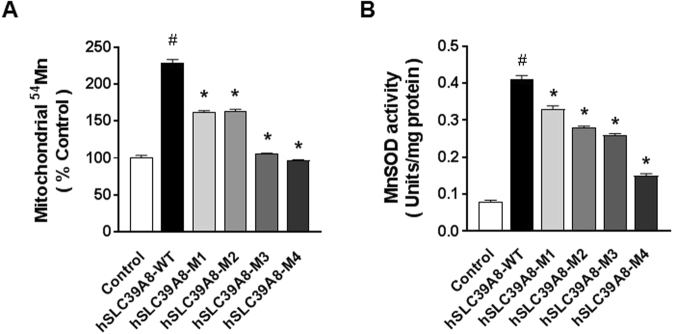
Disease-associated *hSLC39A8* mutants reduce mitochondrial Mn levels and MnSOD activity. (**A**) Mitochondrial Mn levels were measured with ^54^Mn in isolated mitochondria from cells expressing *hSLC39A8*-WT or other mutants. Data represent means ± SEM (n = 3 samples/group). (**B**) MnSOD activity was determined in mitochondria isolated from cells expressing *hSLC39A8*-WT or other mutants. Data represent means ± SEM (n = 3 samples/group). Similar results were obtained in at least three independent experiments. ^#^*P* < 0.05 between control vs. *hSLC39A8*-WT, *P < 0.05 between *hSLC39A8*-WT vs. other mutants; Student’s *t*-test.

Considering the reduction in intracellular Mn levels, together with the association of *SLC39A8* mutations with the mitochondrial disorder Leigh syndrome, we hypothesized that these mutations may lead to reduced activity of MnSOD, the Mn-requiring antioxidant enzyme in mitochondria. We first aimed to determine whether these mutants affect Mn levels in the mitochondria. Mitochondria were isolated in cells expressing *hSLC39A8*-WT or its mutants, and the Mn levels in the mitochondria were directly measured *in vitro* with ^54^Mn. The expression of *hSLC39A8*-WT resulted in a ~2.3-fold increase (*P* < 0.001) in ^54^Mn levels in the mitochondria compared to control cells (Fig. 5A). In contrast, all four mutants displayed significantly lower ^54^Mn levels in the mitochondria compared to WT (*P* < 0.001). These data suggest that the expression of *hSLC39A8*-WT, but not its mutants, provides Mn to the mitochondria.

We next aimed to determine whether the disease-associated mutations affect MnSOD activity. Mitochondria were isolated in cells expressing *hSLC39A8*-WT or its mutants, and MnSOD activity in the mitochondria was measured. Compared to *hSLC39A8*-WT cells, MnSOD activity was significantly reduced in cells expressing *hSLC39A8*-M1 (~19.2%, *P* = 0.008), M2 (~31.4%, *P* = 0.002), M3 (~36.3%, *P* = 0.001), and M4 (~63.3%, *P* < 0.001) (Fig. 5B). Taken together, these results clearly suggest that disease-associated *hSLC39A8* mutations in both CDG type II and Leigh syndrome result in low levels of mitochondrial Mn, which are required for key mitochondrial enzymes, such as MnSOD.

### Disease-associated mutations of *hSLC39A8* negatively influence the expression of genes involved in oxidative phosphorylation

**Figure 6:**
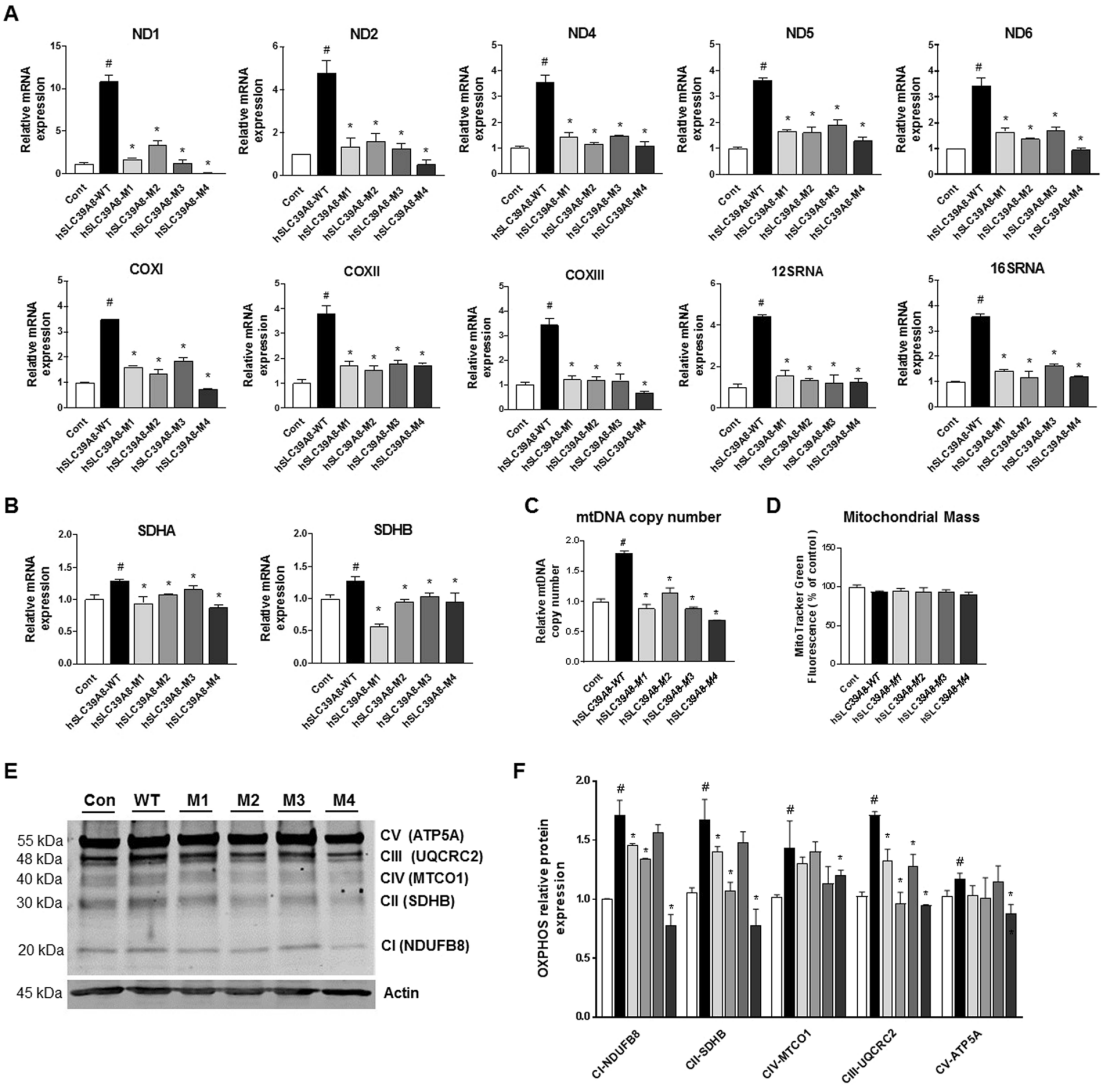
Disease-associated *hSLC39A8* mutants decrease gene expression involved in oxidative phosphorylation. Transcription levels of oxidative phosphorylation genes of (**A**) mtDNA- and (**B**) nuclear DNA-encoded subunits in HeLa cells expressing *hSLC39A8*-WT or its mutants. (**C**) mtDNA copy number and (**D**) mitochondrial mass in HeLa cells expressing *hSLC39A8*-WT or other mutants. (**E**) Representative immunoblot of select electron transport chain protein expression in HeLa cells expressing *hSLC39A8*-WT or other mutants. Equal loading was verified by immunoblotting with actin antibody and the positions of molecular weight markers are shown. Full-length blots are presented in Supplementary Figure 6. (*F*) Quantification of relative select electron transport chain protein expression after normalization with actin. Data represent means ± SEM (n = 2 samples/group). ^#^*P* < 0.05 between control vs. *hSLC39A8*-WT, *P < 0.05 between *hSLC39A8*-WT vs. other mutants; Student’s *t*-test.

While Leigh syndrome is characterized by deficits in mitochondrial functions, mitochondrial abnormality has not been reported in CDG type II. The mitochondrial Mn levels reduced by the *hSLC39A8* mutants associated with both Leigh syndrome (M4) and CDG type II (M1-M3) led us to postulate that these two previously unconnected disorders may share mitochondrial dysfunction as a common feature. We first examined the effect of the mutant expression on mitochondrial function by exploring both mitochondrial DNA (mtDNA)- and nuclear DNA (nDNA)-encoded electron transport genes. For mtDNA genes, we chose *ND1–ND6, COXI– COXIII, 12SRNA*, and *16SRNA*, which are encoded by either the heavy strand or the light strand of mtDNA and known to play important roles in oxidative phosphorylation. Data in Fig. 6A show that the expression of *hSLC39A8*-WT, but not the CDG type II mutants (M1-M3) or the Leigh syndrome mutant (M4), led to increased expression levels of these mtDNA-encoded genes (*P* < 0.05). We further analyzed the mRNA transcript levels of the nDNA-encoded subunits, including succinate dehydrogenase subunits SDHA and SDHB. The gene expression of nDNA-encoded subunits was also significantly reduced in cells expressing each mutant compared to *hSLC39A8*-WT (*P* < 0.05), yet the reduction was generally milder than mtDNA genes (Fig. 6B). We also tested the effect of *hSLC39A8* mutations on the mtDNA copy number. Interestingly, *hSLC39A8*-WT expression, but not its mutants, led to a marked increase in the mtDNA copy number (*P* < 0.05, Fig. 6C), suggesting that *hSLC39A8* can promote the production of mtDNA, but disease-associated mutations interfere with such function. No differences were observed in mitochondrial mass using MitoTracker green in cells expressing *hSLC39A8*-WT or its mutants (Fig. 6D), suggesting that mitochondria in the mutant-expressing cells contain less mtDNA. The reduced mtDNA might be responsible for the reduced transcript levels from mtDNA, at least in part.

We further tested if the levels of mitochondrial electron transport enzymes are altered at the protein level in mutant-expressing cells. Immunoblot analysis was performed using the total OXPHOS antibody cocktail. We found significant increases in levels of the oxidative phosphorylation protein complexes CI, CII, CIII, CIV, and CV in *hSLC39A8*-WT expressing cells (*P* < 0.05) (Fig. 6E,F). The expression levels of CI and CII were significantly reduced in cells expressing *hSLC39A8*-M1, -M2, or -M4 compared to WT (*P* < 0.05). The CIII protein expression level was also significantly decreased in all of the mutants (*P* < 0.05). The expression levels of CIV and CV were significantly inhibited only in *hSLC39A8*-M4 expressing cells. These findings suggest that *hSLC39A8* mutants cannot promote the expression of several electron transport chain proteins as efficiently as WT does. These results suggest that *hSLC39A8* promotes the production of oxidative phosphorylation machineries, and both CDG type II and Leigh syndrome mutations abrogate this function.

### Disease-associated mutations of *hSLC39A8* are linked to impaired mitochondrial function

**Figure 7:**
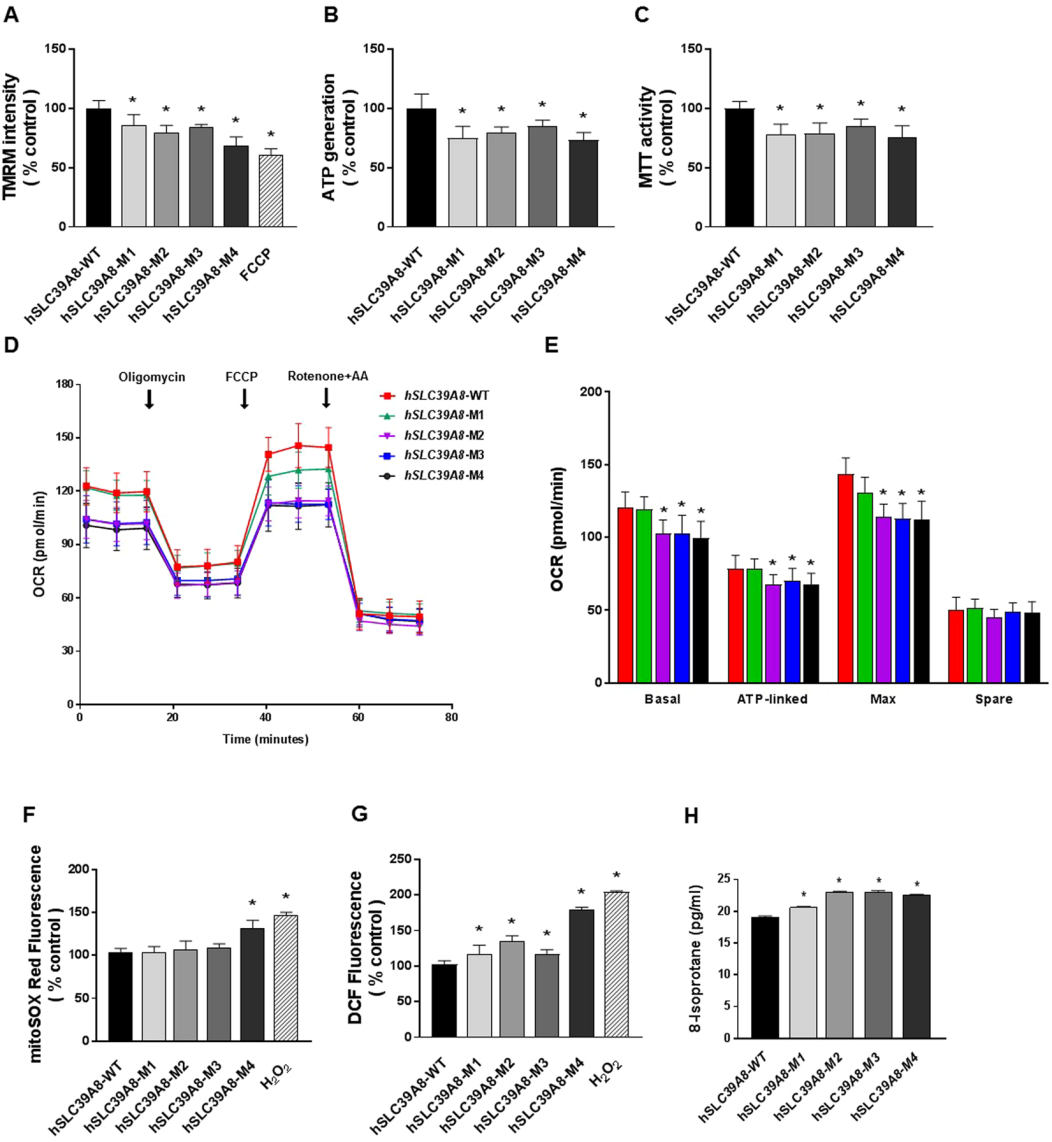
Disease-associated *hSLC39A8* mutants impair mitochondrial function and enhance ROS production. Mitochondrial function and ROS production were assessed in HeLa cells expressing *hSLC39A8*-WT or other mutants. (**A**) The mitochondrial membrane potential was measured by TMRM fluorescence. To validate the assay, the mitochondrial uncoupler FCCP (20 µM) was used as a positive control. (**B**) ATP levels were measured with bioluminescence. (**C**) Metabolic function in the mitochondria was measured with MTT assay. (**D**) Oxygen consumption rate (OCR) as an indication of mitochondrial respiration was performed using the Seahorse XFe96 mitochondrial flux analyzer after the addition of oligomycin (1uM), uncoupling reagent FCCP (0.5uM), and electron transport inhibitor Rotenone/AA (1 uM). (**E**) Basal respiration, ATP turnover, Maximal Respiration, and Spare Mitochondrial capacity were measured as described in the Methods section. (**F**) Mitochondrial superoxide formation was measured with MitoSox Red fluorescence. (**G**) Intracellular ROS formation was measured using DCF fluorescence. To validate the assay, H_2_O_2_ (0.5 mM) was used as a positive control. (**H**) Oxidative damage level was measured with 8-isoprostane in cell supernatant. Data represent means ± SEM as percentage of *hSLC39A8*-WT (n = 5 samples/group). *P < 0.05 vs. *hSLC39A8*-WT; Student’s *t*-test. Similar results were obtained in at least three independent experiments.

The reduced level of oxidative phosphorylation machineries prompted us to test if *hSLC39A8*-mutant expression would affect mitochondrial function. First, we determined the mitochondrial membrane potential (ΔΨ_m_) by tetramethylrhodamine methyl ester perchlorate (TMRM) labeling in cells expressing *hSLC39A8*-WT or its mutants. Because the selective accumulation of cationic dye TMRM in the mitochondria of healthy cells is driven by hyperpolarized mitochondrial membrane potential, the TMRM fluorescence intensity is an indicator of hyperpolarized ΔΨ_m_. Compared to cells expressing *hSLC39A8*-WT, TMRM fluorescence intensity was significantly reduced in cells expressing *hSLC39A8*-M1 (~14.4%, *P* = 0.002), -M2 (~20.5%, *P* < 0.001), -M3 (~16.1%, *P* < 0.001), and -M4 (~32.0%, *P* < 0.001), respectively (Fig. 7A). Next, we performed ATP synthesis assays in the cells. These experiments showed significantly reduced ATP production in cells expressing *hSLC39A8*-M1 (~25.1%, *P* < 0.001), -M2 (~20.4%, *P* < 0.001), -M3 (~14.9%, *P* < 0.001), and -M4 (~26.6%, *P* < 0.001), respectively, compared to *hSLC39A8*-WT cells (Fig. 7B). Moreover, compared to cells expressing *hSLC39A8*-WT, mitochondrial redox activity measured by MTT assay was significantly reduced in cells expressing *hSLC39A8*-M1 (~22.0%, *P* < 0.001), -M2 (~21.3%, *P* < 0.001), -M3 (~14.9%, *P* < 0.001), and -M4 (~24.6%, *P* < 0.001), respectively (Fig. 7C).

Because oxidative metabolism is an important function of mitochondria, we measured the mitochondrial respiration using a Seahorse extracellular flux analyzer. We found a significant decrease in basal respiration, ATP-linked respiration, and maximal respiration in cells expressing *hSLC39A8*-M2, -M3, and -M4, respectively, compared to *hSLC39A8*-WT (Fig. 7D,E). Taken together, these results indicate that compared to the WT, *hSLC39A8* mutants are less capable of supporting mitochondrial functions, including mitochondrial membrane potential, ATP production, mitochondrial redox activity, and mitochondrial respiration.

### The expression of *hSLC39A8* mutants enhances ROS generation

The mitochondrial electron transport chain is the major intracellular source of reactive oxygen species (ROS), including superoxide anion, hydroxyl radical, and various peroxides and hydroperoxides^20^. Scavenging ROS is an important role of mitochondria^21^. To determine whether the observed mitochondrial dysfunction also involves impaired ROS scavenging, we measured ROS levels using fluorescent indicators specifically for superoxide (O^2−^) (MitoSox Red) and H_2_O_2_ production (H_2_DCFDA). MitoSOX Red selectively targeted to the mitochondria exhibits red fluorescence following oxidation by superoxide anion, thereby serving as an indicator of mitochondrial superoxide levels. Compared to cells expressing *hSLC39A8*-WT, MitoSOX Red fluorescence intensity was significantly increased in cells expressing *hSLC39A8*-M4 by ~30.4% (*P* < 0.001), while *hSLC39A8*-M1, M2, and M3 were not observed to have an effect (Fig. 7F). We then measured global oxidative stress level by H_2_DCFDA, which is converted to highly fluorescent 2′,7′-dichlorofluorescein (DCF) following the removal of the acetate groups by intracellular esterases and ROS-induced oxidation. The DCF fluorescence was significantly increased in cells expressing *SLC39A8* M1 (~16.4%, *P* < 0.05), M2 (~35.0%, *P* < 0.001), M3 (~16.4%, *P* = 0.006), and M4 (~79.8%, *P* < 0.001) compared to cells expressing *hSLC39A8*-WT (Fig. 7G). Furthermore, we measured the oxidative damage levels using 8-isoprostane levels. Our data indicated that cells expressing each of the four *hSLC39A8* mutants significantly increased isoprostane levels compared to *hSLC39A8*-WT cells (*P* < 0.001). These results indicate that disease mutations interfere with *hSLC39A8* function to suppress excess ROS levels with a stronger impact of Leigh syndrome associated mutation (M4) within the mitochondria.

### Knockdown of *hSLC39A8* reduces Mn uptake activity and impairs mitochondrial function

**Figure 8:**
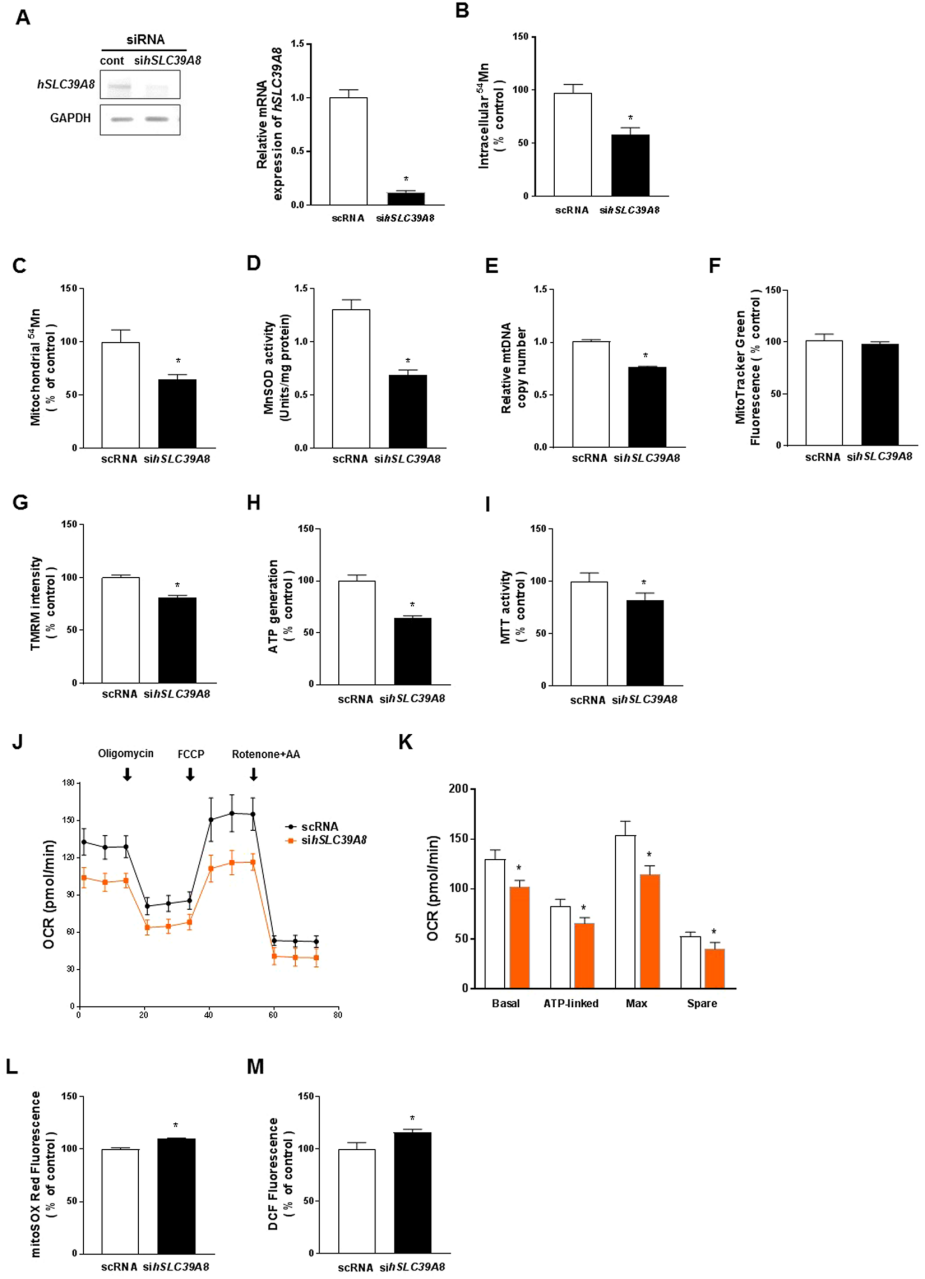
Loss of *hSLC39A8* abrogates ^54^Mn uptake activity and mitochondrial function. (**A**) HeLa cells were transfected with control or anti-*hSLC39A8* siRNAs. Down-regulation of si*hSLC39A8* was confirmed by semi-quantitative RT-PCR 48 h after the transfection of each siRNA. Effects of *hSLC39A8* knockdown on intracellular ^54^Mn (**B**), Mitochondrial ^54^Mn (**C**), MnSOD activity (**D**), the mtDNA copy number (**E**), mitochondrial mass by MitoTracker Green fluorescence (**F**), mitochondrial membrane potential by TMRM fluorescence (**G**), ATP levels by bioluminescence (**H**), mitochondrial redox activity by MTT assay (**I**), OCR as an indication of mitochondrial respiration by the Seahorse XFe96 mitochondrial flux analyzer (**J** and **K**), mitochondrial superoxide formation by MitoSox Red fluorescence (**L**), and intracellular ROS formation by DCF fluorescence (**M**). Data represent means ± SEM (n = 3–5 samples/group). *P < 0.05 vs. control siRNA (scRNA)-transfected cells; Student’s *t*-test.

In addition to the effects of *hSLC39A8* overexpression, we tested the effect of *hSLC39A8* depletion on Mn uptake and mitochondrial function. We used siRNA to knockdown *hSLC39A8* in HeLa cells. RT-PCR analysis showed that *hSLC39A8* is effectively reduced by ~90% (*P* < 0.001) compared with cells transfected with a control siRNA that did not target any human gene (Fig. 8A). Our data indicated that the knockdown of *hSLC39A8* reduced ^54^Mn uptake activity by ~45% (*P* < 0.001) compared to control siRNA-transfected cells (Fig. 8B). Furthermore, ^54^Mn levels in the mitochondria (~35%, *P* < 0.001) and MnSOD activity (~47%, *P* < 0.001) in *hSLC39A8*-depleted cells were significantly reduced (Fig. 8C,D). We also found a significant reduction in the mtDNA copy number in *hSLC39A8*-depleted cells (~24%, *P* = 0.002) (Fig. 8E), with no significant change in mitochondrial mass (Fig. 8F). Importantly, *hSLC39A8* RNAi significantly reduced mitochondrial membrane potential (~20%, *P* < 0.001), ATP production (~37%, *P* < 0.001), and mitochondrial redox activity (~20%, *P* < 0.001). We also measured the mitochondrial respiration using the Seahorse extracellular flux analyzer, and found a significant decrease in basal respiration, ATP-linked respiration, maximal respiration, and spare respiratory capacity in *hSLC39A8*-depleted cells compared with control siRNA cells (Fig. 8J,K). Concordantly, the knockdown of *hSLC39A8* significantly increased superoxide production by MitoSox Red (~10%, *P* < 0.001) and H_2_O_2_ production by H_2_DCFDA (~16%, *P* < 0.001). Taken together, the knockdown results complement our overexpression studies and indicate that *hSLC39A8* plays an essential role in mediating Mn uptake and modulating mitochondrial function.

## Discussion

In this report, we describe the first *in vitro* functional analysis of human *SLC39A8* mutations associated with severe Mn deficiency^8,9,11^. Our studies have led to several insights regarding the function and regulation of human *SLC39A8* as a Mn transporter, and shed novel light on the mechanisms by which mutations in *hSLC39A8* cause Mn deficiency-associated developmental conditions.

First, we provided several lines of evidence for the specific requirement of *hSLC39A8* for Mn uptake among other metals, such as zinc. Our radiolabeled ^54^Mn uptake studies have shown that *hSLC39A8* has a high affinity for Mn. The *Km* of ~1.44 ± 0.39 µM for Mn^2+^ is consistent with previous reports on *SLC39A8*^5,7^ and other mammalian SLC39 transporters^13,14^ and within a similar range observed in other cell lines or tissues^5,7,13,14^. Our metal competition studies further confirm that *hSLC39A8* has a higher affinity for Mn and cadmium compared to zinc. Previous *in vitro* studies have shown that *SLC39A8* mediates the uptake of zinc, iron, and cadmium^5,6^. In the competition assay, we also found that zinc could compete with Mn, yet to a lesser extent compared to Mn and Cd. The ability of *hSLC39A8* to transport zinc or the competition by zinc in these assays may not necessarily mean that this protein physiologically transports zinc. This idea is supported by the fact that unlike *in vitro* studies^5,6^, human patients carrying *hSLC39A8* mutations^8,9,11^ or *SLC39A8*-inducible global and liver-specific knockout mice^7^ have shown no changes in zinc and iron levels. Furthermore, in our experiments, the difference in metal transport between *hSLC39A8*-WT and the four disease mutants was only observed for Mn, not for zinc, iron, or copper (Supplementary Fig. 2). Based on these observations, it is likely that *hSLC39A8* acts primarily as a Mn transporter under physiological conditions. However, we cannot rule out the possibility that *hSLC39A8* may be necessary for transporting zinc under certain circumstances, such as when cells lack other efficient zinc transporters.

The mislocalization of pathogenic mutations in *SLC39A8* to the ER (Fig. 4) suggests that these mutations may alter protein targeting motifs and/or lead to loss-of-function of its intrinsic metal transport activity. As shown in Fig. 2A,B, topology prediction of *hSLC39A8* reveals that *hSLC39A8* adopts the classic SLC39 structure of eight transmembrane (TM) domains with their N- and C- termini facing the extracytoplasmic space^4^. Disease-associated mutations reside in either the N-terminal cytoplasmic region, TM III, or TM IV. Importantly, these mutations change non-polar residues (Glycine, Isoleucine, Valine, and Cysteine) to polar (Asparagine, Threonine) or charged (Arginine) amino acids that are likely to disrupt protein folding. All four mutations found in the N-terminal cytoplasmic regions might affect the sequence motifs that control their subcellular localization, trafficking, and function. Further studies will be required to test whether mutations in these distinct protein segments influence Mn transport activity.

Mn is an essential nutrient that acts as a cofactor for key biological enzymes, including carboxylases and phosphatases in the cytosol, sugar transferase in the Golgi, and superoxide dismutase in mitochondria^22–25^. Despite its essentiality, excess Mn accumulates in the mitochondria^26,27^, producing Mn toxicity associated with mitochondrial dysfunction, oxidative stress, and cell apoptosis^28,29^. While a number of studies have shown the effect of excess Mn on mitochondrial function, our study demonstrates that Mn deficiency caused by *hSLC39A8* deficiency may impair mitochondrial function and enhance oxidative stress. The expression of both CDG type II-associated *hSLC39A8* mutants (*hSLC39A8*-M1, -M2, -M3) and the mitochondrial disorder-associated *hSLC39A8* mutant (*hSLC39A8*-M4) resulted in mitochondrial dysfunction and oxidative stress. Glycosylation defects in CDG type II are thought to be explained by the requirement of Mn for the activity of β-1,4-glycosyltransferase^9^. It is currently unknown whether these patients have mitochondrial abnormalities. Testing if CDG type II also involves abnormalities of mitochondria, including their morphology, dynamics, and function, will likely provide important insights into the pathogenesis of the developmental and neurological symptoms observed in this condition.

The novel role of *hSLC39A8* in mitochondrial functions may involve multiple mechanisms. Mn is required as a cofactor for MnSOD, a reactive oxygen species scavenger found in mitochondria^30^. Mn deficiency in yeast leads to the reduced activity of MnSOD and elevated levels of superoxide^31^. ROS can damage enzymes containing Fe-S clusters, including complex I, II, and III of the respiratory chain, and damage mtDNA, which encodes subunits of complex I, III, IV, and V^30^. Our data clearly demonstrate that the expression of *hSLC39A8* mutants reduced mitochondrial Mn levels and MnSOD activity concurrent with increased ROS levels. Thus, we speculate that reduced MnSOD activity by *hSLC39A8* mutants generates ROS, which may negatively influence oxidative phosphorylation machineries and impair mitochondrial function. Additional mechanisms of *hSLC39A8*-mediated enhancement of mitochondrial functions could be promoting the expression of oxidative phosphorylation-related genes and maintaining the appropriate mtDNA copy number (Fig. 6). Again, altered mitochondrial ROS might underlie the change in the mtDNA copy number. Future studies are required to examine the mechanistic relationship between intracellular Mn levels and mitochondrial function.

The *hSLC39A8*’s role in providing Mn to MnSOD to suppress excess ROS may provide a hint regarding the pathogenesis of neurological symptoms observed in human patients. All patients with *hSLC39A8* mutations show intellectual disability and cerebellar atrophy^8,9,11^. Recent epidemiological studies have shown that both low and high Mn exposures are associated with adverse neurodevelopmental outcomes^32,33^. MnSOD knockout mice showed neuronal degeneration in the basal ganglia and brainstem that was characterized by extensive mitochondrial damage^34^. Similar clinical pathologies, such as progressive neurodegeneration with brainstem and basal ganglia dysfunction, are observed in the *SLC39A8*-deficienct patients with Leigh-like mitochondria diseases^35^. However, we note the limitation of our study as well. *SLC39A8* is widely expressed in many tissue cell types^36^. Studies are currently underway to validate the roles of *SLC39A8* in other cell types, especially the ones related to neurological symptoms, and should be able to define the causal roles of Mn, ROS, and mitochondria in neurological symptoms that are linked to *SLC39A8*.

**Figure 9:**
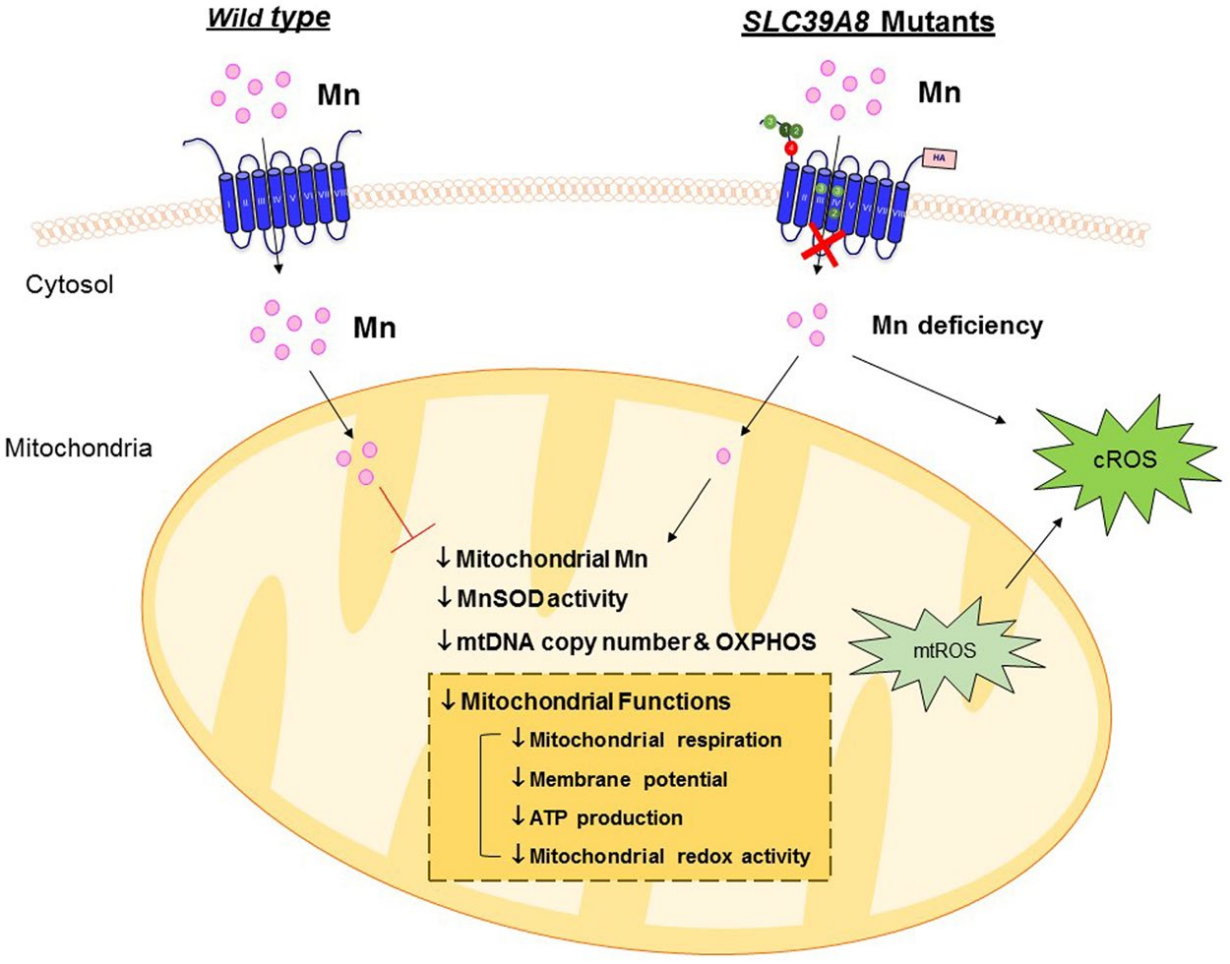
Scheme of the influence of *hSLC39A8* deficiency on Mn uptake and mitochondrial function. *SLC39A8* deficiency results in reduced intracellular Mn entry and mitochondrial Mn levels. Decreased intracellular Mn results in reduced MnSOD activity, as well as decreased mitochondrial DNA copy number and decreased expression of mitochondrial genes. Decreased mitochondrial Mn, together with the decreased expression of mitochondrial genes, impairs mitochondrial function including mitochondrial respiration, mitochondrial membrane potential, ATP production, and mitochondria redox activity, which subsequently results in a rise in increased ROS generation. Decreased MnSOD activity further results in increased ROS.

In summary, our current study provides direct and functional evidence that *hSLC39A8* is a Mn-specific transporter and genetic mutations in *hSLC39A8* interfere with Mn uptake and mitochondrial function (Fig. 9). Concomitantly, the expression of these mutants and depletion of *hSLC39A8* by RNAi reduces the mitochondrial Mn levels, MnSOD activity, and gene expression of oxidative phosphorylation enzymes, which are accompanied by mitochondrial dysfunction and increased oxidative stress. Our studies provide an important link between Mn and mitochondrial function in the pathogenesis of diseases that are associated with *hSLC39A8*. The findings extend our current knowledge of the pathogenesis of inherited Mn deficiencies, which may facilitate the development of therapeutic targets to treat these disorders. Further studies are warranted to evaluate the incidence of these mutations in the human population and their relevance to human health and disease.

## Methods

### Vector Construction and Site-directed Mutagenesis of *hSLC39A8*

*hSLC39A8* cDNA was obtained from a cDNA clone (GenBank^TM^ accession number BC012125.1). The entire coding region of the wild-type forms of *hSLC39A8* were amplified and tagged at the C terminus with two tandem hemagglutinin (HA) epitopes by PCR using primers (FWD 5′-CCACCATGGCCCCGGGTCGCGCGGTG-3′ and Rev 5′-GGCGTAGTCGGGGACGTCGTAGGGGTAGGCGTAGTCGGGGACGTCGTAGGGGTACTCCAATTCGATTTCTCCTGC-3′ (25 cycles: 94 °C for 30 s, 58 °C for 30 s, and 72 °C for 2.5 min) and inserted into pcDNA3.1/V5-His TOPO vector (Invitrogen). Point mutations were introduced into this construct using the QuikChange mutagenesis kit (Agilent Technologies). The site-directed mutation, orientation and fidelity of the insert, and incorporation of the epitope tag were confirmed by directed sequencing (University of Michigan DNA Sequencing Core). The sequencing data and the plasmids are available upon request.

### Cell culture and the expression of *hSLC39A8* and its mutants in HeLa cells

All culture media and supplements were purchased from Invitrogen (Carlsbad, CA, USA). Heat-inactivated fetal bovine serum was purchased from Sigma-Aldrich (St. Louis, MO, USA). HeLa cells were grown in DMEM containing 10% fetal bovine serum, penicillin (100 IU/ml), and streptomycin (100 mg/ml) at 37 °C in a humidified, 5% CO_2_ incubator. DNA transfections were performed with Lipofectamine 3000 (Invitrogen) according to the manufacturer’s specifications. Cultures were generally transfected 24 h after plating and used 48 h after transfection. For small interfering RNA-mediated gene suppression of *SLC39A8*, HeLa cells were plated in six-well plates overnight and then transfected with 50 nM of control siRNA (Sigma, SIC001–10NMOL) or *hSLC39A8* siRNA (Sigma, SASI_Hs02_00355573) using Lipofectamine 3000 (Invitrogen) as described above.

### Immunofluorescence and microscopy

For confocal studies, transfected cells were plated onto glass coverslips and were fixed with 4% paraformaldehyde in phosphate-buffered saline (PBS) for 10 min, and immunofluorescence staining was performed as described previously^18,37^. To permeabilize the cells, cells were incubated with 0.2% Triton X-100 in PBS for 5 min. Nonspecific binding was blocked with 4% bovine serum albumin (BSA) in PBS for 30 min, and transfected HA-tagged *hSLC39A8***-**WT or its mutants were detected following incubation with Alexa 488-conjugated anti-mouse HA (1 μg/ml, Biolegend, Cat no. 90159) for 1 h. Rabbit anti-calnexin (1 μg/ml, Abcam, Cat no. ab22595) or pan-cadherin (1 μg/ml, Abcam, Cat no. ab6529) was used as ER or cell surface markers, respectively. Detection of calnexin or pan-cadherin was performed using an anti-rabbit IgG antibody conjugated to Alexa 568 (1 μg/ml, Invitrogen, Cat no. A11036) for 20 min. Coverslips were drained, mounted in ProLong Gold (Invitrogen), and sealed with nail polish. Immunofluorescent imaging was performed using a Nikon Eclipse A-1 confocal microscope (Nikon, Melville, NY, USA) with a 60× oil immersion lens. Colocalization analysis was performed after background subtraction through the use of the colocalization function in Fiji by ImageJ, and colocalized pixels were pseudocolored yellow. To quantify the subcellular localization of each mutant with calnexin or pan-cadherin, statistical analysis of the correlation of the intensity values of green and red pixels in a dual-channel image was performed using a correlation coefficient [Pearson’s coefficient (PC)]. The value can range from +1 to −1, with +1 illustrating a positive correlation, −1 a negative correlation, and zero illustrating no correlation^19^.

### Quantitative RT-PCR

Total RNA was extracted from cells using TRIzol reagent (Invitrogen) as per the manufacturer’s instructions. RNA was purified and on-column DNAse treated using the Direct-zol RNA Miniprep Kit from Zymo Research (Irvine, CA) following the manufacturer’s instructions. Purified RNA was then reverse-transcribed with SuperScript^®^ III First-Strand Synthesis System (Invitrogen). Quantitative PCR (qPCR) was performed using Power SYBR-Green PCR Master Mix (Applied Biosystems). 18SRNA and nDNA were used for normalization of mRNA and mtDNA, respectively. The primers used for qPCR are listed in Table 1 and were all purchased from Integrated Genomics Technologies.

**Table 1:** Primer sequences used in this study.

*Gene*	*Forward primer*	*Reverse primer*
*SDHA*	CCTTTCTGAGGCAGGGTTTA	AGAGCAGCATTGATTCCTCC
*SDHB*	ACCTTCCGAAGATCATGCAGA	GTGCAAGCTAGAGTGTTGCCT
*PGC-1α*	TGAGAGGGCCAAGCAAAG	ATAAATCACACGGCGCTCTT
*NRF1*	GGGAGCTACAGTCACTATGG	TCCAGTAAGTGCTCCGAC
*NRF2*	TACTCCCAGGTTGCCCACA	CATCTACAAACGGGAATGTCTGC
*TFAM*	CCGAGGTGGTTTTCATCTGT	TCCGCCCTATAAGCATCTTG
*18S RNA*	GAGGTAGTGACGAAAAATAACAAT	TTGCCCTCCAATGGATCCT
*ND1*	CCTAGGCCTCCTATTTATTC	GAATGATGGCTAGGGTGA
*ND2*	CTACGCCTAATCTACTCCAC	CTTTGAAGGCTCTTGGTCTG
*ND4*	GGACTCCACTTATGACTCCC	GGTTGAGAATGAGTGTGAGGC
*ND5*	CTATCACCACTCTGTTCGCAG	GTGGTTGGTTGATGCCGATTG
*ND6*	CTAAAACACTCACCAAGACC	GGAATGATGGTTGTCTTTGG
*COXI*	GATTTTTCGGTCACCCTGAAG	CTCAGACCATACCTATGTATC
*COXII*	CTATCCTGCCCGCCATCATC	GATTAGTCCGCCGTAGTCGG
*COXIII*	CACATCCGTATTACTCGCATC	GAAGTACTCTGAGGCTTGTAG
*12SRNA*	CACTACGAGCCACAGCTTAA	TCAGGGTTTGCTGAAGATGG
*16SRNA*	GGCATGCTCATAAGGAAAGG	GGCCGTTAAACATGTGTCAC
*β-globin*	CCTTTGTTCCCTAAGTCCAA	CCTCACCTTCTTTCATGGAG

### MtDNA copy number

Total DNA was extracted from cell samples via TRIzol (Invitrogen) extraction. Following complete removal of the RNA-containing aqueous phase, DNA extraction buffer [Tris base (1 M), sodium citrate dibasic trihydrate (50 mM), and guanidine thiocyanate (4 M)] was added to the tubes containing the remaining Trizol-separated interphase and infranatant. The tubes were shaken vigorously and centrifuged at 12,000 × *g* at room temperature for 30 min. The aqueous phase was collected, and the genomic and mitochondrial DNA were precipitated in isopropanol. Samples were respun at 12,000 × *g* at 4 °C to pellet the DNA. The DNA pellet was then washed in 70% ethanol, respun, and, after careful ethanol removal, resuspended in TE buffer. To quantify the mtDNA copy number, qPCR was performed as described above against external standards for mtDNA and β-globin using primers listed in Table 1.

### MitoTracker assay

The Mitochondrial mass was measured by a MitoTracker Green FM dye (Invitrogen), a dye that stains mitonchondria independent of its membrane potential. Cells were stained with 100 nM MitoTracker probes at 37 °C for 30 min and washed two times with PBS. The fluorescence of MitoTracker (excitation 495 nm, emission 520 nm) was measured at 25 °C using a BioTek Synergy microplate reader (BioTek Instruments, Winooski, VT).

### Immunoblot analysis

The total lysates were prepared in a hypotonic buffer using 1% NP-40 plus protease inhibitors (Roche, Cat. No. 11836153001). Protein concentration was determined by Bradford assay. Samples (30–50 μg) were separated by electrophoresis and transferred to a nitrocellulose membrane (Bio-Rad, Cat. No. 1620115). The membrane was immunoblotted with anti-mouse HA antibody (Biolegend, Cat. No. 901501), anti-rabbit GPP130 antibody (Biolegend, Cat. No. 923801), anti-rodent total OXPHOS antibody (Abcam, Cat. No. ab110413), anti-mouse GFP antibody (Santa Cruz, Cat. No. 101536), and anti-mouse actin (Proteintech, Cat. No. 60008–1-Ig). The blots were visualized with infrared anti-mouse or anti-rabbit secondary antibodies, using a LI-COR Odyssey fluorescent Western blotting system (LI-COR Biosciences). Protein expression was quantified using densitometry (Image Studio Lite; LI-COR).

### Mn superoxide dismutase assay

Mitochondrial MnSOD activity was determined using a Superoxide Dismutase Assay Kit (Cayman Chemical, Ann Arbor, MI, USA) as described previously^38^. Briefly, the cells were scraped into ice-cold MB buffer (10 mM HEPES, pH 7.5, 210 mM mannitol, 70 mM sucrose, 1 mM EDTA) and then dounce homogenized with a glass homogenizer. The cell extract was centrifuged at 1,000 × g for 5 min at 4 °C, and the mitochondrial fraction was pelleted by centrifugation at 12,000 × g for 12 min at 4 °C. MnSOD activity was determined in the presence of 2 mM potassium cyanide to inhibit Cu/Zn-SOD.

### ^54^Mn uptake assay

^54^Mn uptake assay was determined as described previously^39,40^. Briefly, cells were incubated at 37 °C for 30 min in serum-free media containing 1 µM ^54^Mn. Cells were then chilled on ice and washed three times with PBS containing 1 mM EDTA to remove any unbound ^54^Mn. Cell-associated radioactivity was determined with a gamma counter and was normalized to the cell protein measured in lysates using the Bradford assay. For mitochondrial ^54^Mn levels, mitochondria were isolated through differential centrifugation, and then mitochondria-associated ^54^Mn levels were measured with a gamma counter.

### Trace element analysis

HeLa cells transfected with *hSLC39A8*-WT or other mutants were analyzed for metals by inductively coupled plasma mass spectrometry (ICP-MS) (Lumigen Instrument Center, Department of Chemistry, Wayne State University, MI, USA), as described previously^38,41^. Briefly, the cell samples were digested with 2 mL/g total wet weight nitric acid (BDH ARISTAR^®^ULTRA) for 24 h, and then digested with 1 mL/g total wet weight hydrogen peroxide (BDH Aristar^®^ ULTRA) for 24 h at room temperature. The samples were preserved at 4 °C until quantification of metals. Ultrapure water was used for final sample dilution.

### Monitoring of mitochondrial membrane potential (ΔΨm)

The mitochondrial membrane potential was measured by TMRM (Invitrogen). Red fluorescence served as the indicator for mitochondrial membrane potential. Briefly, cells were loaded with 50 nM TMRM at 37 °C for 30 min and washed two times with PBS. The fluorescence of TMRM (excitation 548 nm, emission 574 nm) was measured at 25 °C using a BioTek Synergy microplate reader (BioTek Instruments, Winooski, VT). Cells were treated with carbonilcyanide p-triflouromethoxyphenylhydrazone (FCCP, 20 µM) for 10 min as a positive control.

### MTT mitochondrial redox activity assay

Mitochondrial metabolic function was assessed using an MTT assay, as described previously^38,39^. This assay is based on the ability of the mitochondrial enzyme succinate dehydrogenase to metabolize MTT into formazan. The cells were incubated for 3 h with MTT (0.5 mg/ml), and then MTT solvent (isopropyl alcohol containing 0.1 N HCl) was added to dissolve the formazan formed. The resulting formazan was quantified spectrophotometrically at 570 nm, and the background was subtracted at 690 nm using a plate reader.

### Assessment of ATP production rate

The ATP production rate was measured by a CellTiter-Glo® Luminescence Cell Viability Assay kit (Promega) according to the manufacturer’s instruction. The amount of ATP was directly proportional to the number of living cells present in the culture. Briefly, cells expressing *hSLC39A8*-WT or its mutants were cultured in 96-well white-walled plates. Then, 100 µl of CellTiter-Glo reagent was added to lyse the cells. The contents were mixed for 2 min on an orbital shaker to induce cellular lysis followed by incubation at room temperature for 10 min to stabilize the signal; the luminescence was then recorded immediately. The luminescence intensity was measured at 25 °C using a plate reader.

### Analysis of mitochondrial respiration

Extracellular flux analyses were performed using a Seahorse XFe 96 analyzer (Agilent Technologies, Santa Clara, CA, USA), according to the manufacturer’s instructions. Twenty-four hours before assay, HeLa cells transfected with *hSLC39A8*-WT or its mutants, *hSLC39A8*-siRNA, or scrambled siRNA, were cultured on Seahorse XF96 plates at a density of 8 × 10^3^ cells per well. Cells were washed and incubated with XF assay Medium (Seahorse Bioscience), and supplemented with 25 mM glucose, 1 mM sodium pyruvate, and 2 mM L-glutamine at 37 °C and 0% CO_2_ for 1 hour. Baseline oxygen consumption rate (OCR) were measured at 37 °C four times before sequentially injecting the following: Oligomycin (1 uM) to measure the ATP-linked OCR, oxidative phosphorylation uncoupled FCCP (0.5 uM) to determine maximal respiration, and rotenone (1 uM) and antimycin A (1uM) to determine the non-mitochondrial respiration. OCR were automatically calculated by the Seahorse XFe-96 software.

### Measurement of Intracellular and Mitochondrial ROS formation

ROS levels were measured using fluorescent indicators specific for superoxide (O^2−^) (MitoSox Red) and H_2_O_2_ production (H_2_DCFDA). MitoSox Red selectively targeted to the mitochondria is oxidized by superoxide and exhibits red fluorescence. Cells were incubated with 5 μM MitoSOX Red (Invitrogen) for 30 min at 37 °C. The fluorescence of MitoSOX (excitation 510 nm, emission 590 nm) was measured at 25 °C using a BioTek Synergy HTX Multi-Mode Microplate Reader (BioTek Instruments, Winooski, VT). H_2_DCFDA, a cell permeant ROS indicator, is nonfluorescent and then converted to highly fluorescent 2′,7′-dichlorofluorescein (DCF) following the removal of the acetate groups by intracellular esterases and ROS-induced oxidation. Cells were incubated with 3 μM DCFH-DA (Invitrogen) for 30 min at 37 °C. Fluorescence of DCF (excitation 495 nm, emission 520 nm) was measured at 25 °C using a plate reader. Cells treated with hydrogen peroxide (H_2_O_2_, 0.5 mM) for 30 min were used as a positive control.

### Isoprostane analysis

Isoprostane was chosen as a marker of oxidative damage, and measured by a competitive enzyme-linked immunosorbent assay (ELISA) for one of the isoprostanes 8-iso Prostaglandin F_2α_ (8-iso-PGF) with a commercial kit (Cayman Chemical, Ann Arbor, MI). The assay was based on the competition between 8-iso-PGF and 8-isoprostane-acetylcholinestase (AChE) conjugate for a limited number of binding sites in each ELISA plate well. The concentration of 8-iso-PGF is inversely proportional to the number of binding sites available, whereas AChE is held constant. The assays were performed in accordance with the manufacturer’s instructions.

### Statistical analysis

Statistical comparisons were determined with a Student’s t-test using Prism 7 software (GraphPad Software) for all statistical analyses. Values of *P* < 0.05 were considered statistically significant. Asterisks in graphs, wherever present, denote statistically significant differences.

## Electronic supplementary material

Supplementary information
