# Supplementary material for: Functional analysis of SLC39A8 mutations and their implications for manganese deficiency and mitochondrial disorders
Source: Sci Rep. 2018 Feb 16;8:3163. doi: 10.1038/s41598-018-21464-0 (PMC5816659; doi:10.1038/s41598-018-21464-0)
Supplement: Supplementary file 1 — Supplementary information [file 41598_2018_21464_MOESM1_ESM.docx]

**Supplementary Information**

**Functional analysis of *SLC39A8* mutations and their implications for manganese deficiency and mitochondrial disorders**

Eun-Kyung Choi^1^, Trang-Tiffany Nguyen^1^, Neil Gupta^1^, Shigeki Iwase^2^, and Young Ah Seo^1^*

^1^Department of Nutritional Sciences, University of Michigan School of Public Health, MI 48109 USA

^2^Department of Human Genetics, University of Michigan, MI 48109, USA

* Correspondence and requests for materials should be addressed Y.A.S (email: [youngseo@umich.edu](mailto:youngseo@umich.edu))

**TABLE OF CONTENTS**

**Supplemental Figure 1. GPP130 is degraded in lysosomes in the cells expressing *hSLC39A8*.**

**Supplemental Figure 2.** **Disease-associated *hSLC39A8* mutations did not alter intracellular levels of zinc, iron, and copper.**

**Supplementary Figure 3. Full-length immunoblot images in Figure 1E.**

**Supplementary Figure 4. Full-length immunoblot images in Figure 3C.**

**Supplementary Figure 5. Full-length immunoblot images in Figure 3E.**

**Supplementary Figure 6. Full-length immunoblot images in Figure 6E.**

**Supplementary Figure 7. Full-length immunoblot images in Supplementary Figure 1.**


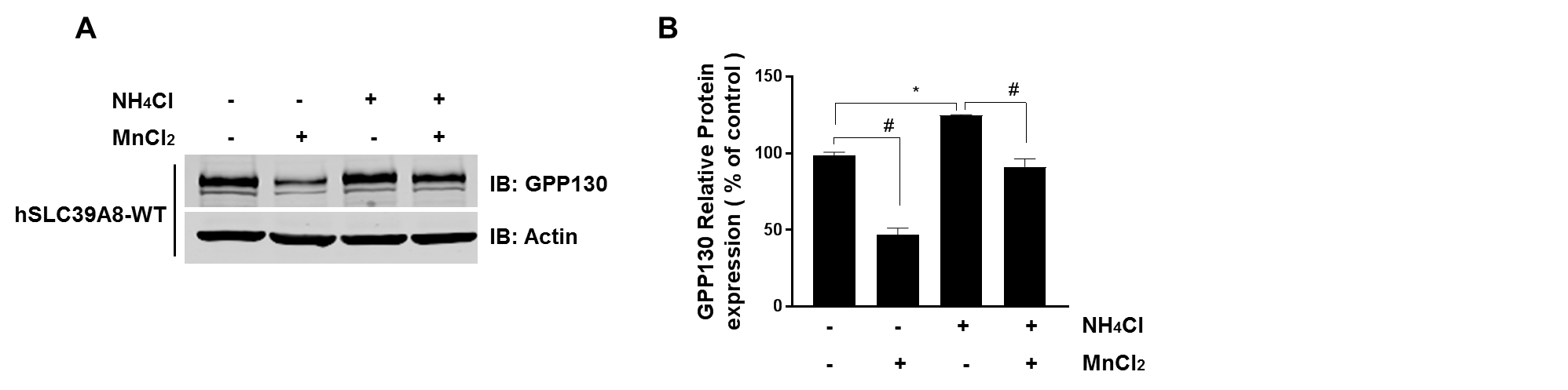


**Supplemental Figure 1. GPP130 is degraded in lysosomes in the cells expressing *hSLC39A8*.** (A) Representative immunoblot of GPP130 in total cell lysates isolated from *hSLC39A8*-WT-expressing cells treated with 500 µM MnCl_2_ in the presence and absence of the lysosome inhibitor NH_4_Cl. Equal loading was verified by immunoblotting with actin antibody. Full-length blots are presented in Supplementary Figure 7. (B) Quantification of GPP130 relative protein after normalization with actin. Results are means ± SEM of three independent experiments. ^#^ *P <* 0.05 *vs.* control in the Mn treatment, * *P* < 0.05 *vs.* control in the NH_4_Cl treatment; Student’s *t*-test.

**
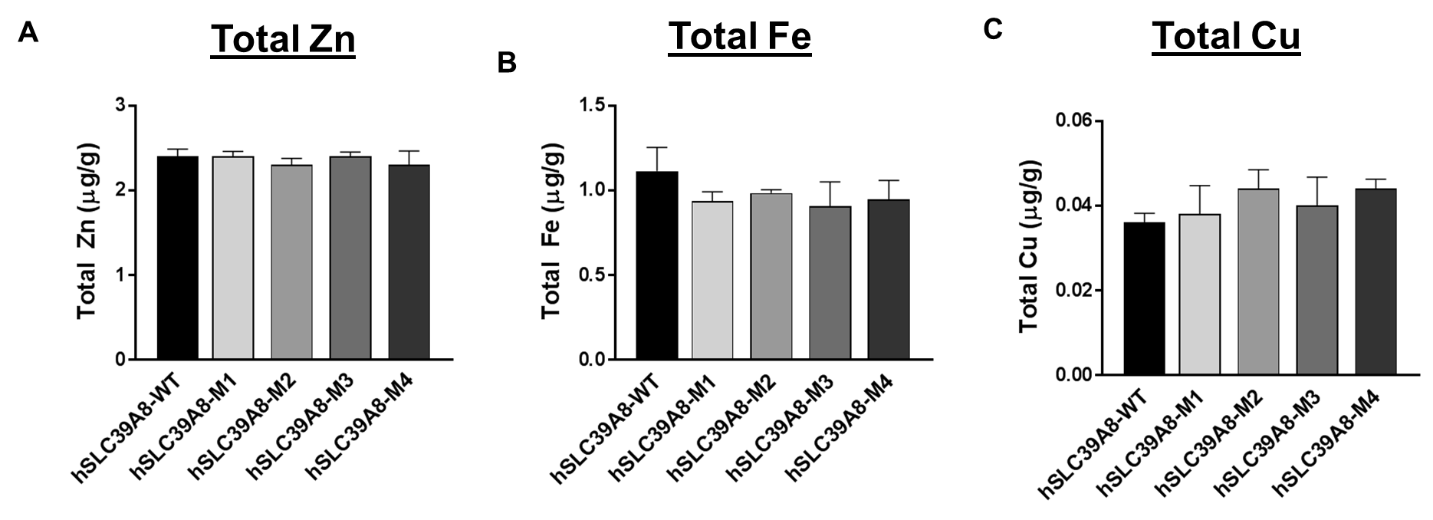
Supplemental Figure 2.** **Disease-associated *hSLC39A8* mutations did not alter intracellular levels of zinc, iron, and copper.** HeLa cells were transfected with *hSLC39A8*-WT or *hSLC39A8* mutants for 48 h. Intracellular metal levels including zinc (A), iron (B), and copper (C) were measured by ICP-MS. Data represent means ± SEM (n = 3 samples/group).

**
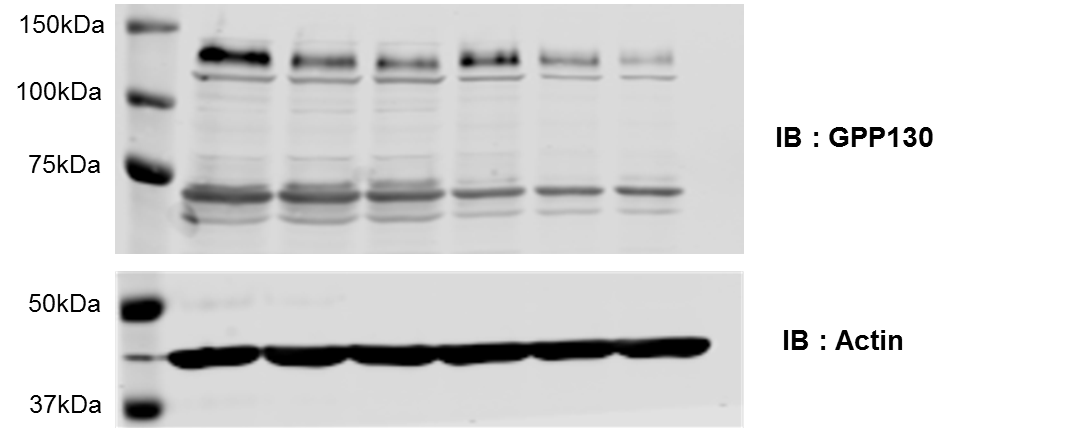
**

**Supplementary Figure 3. Full-length immunoblot images in Figure 1E.**

**
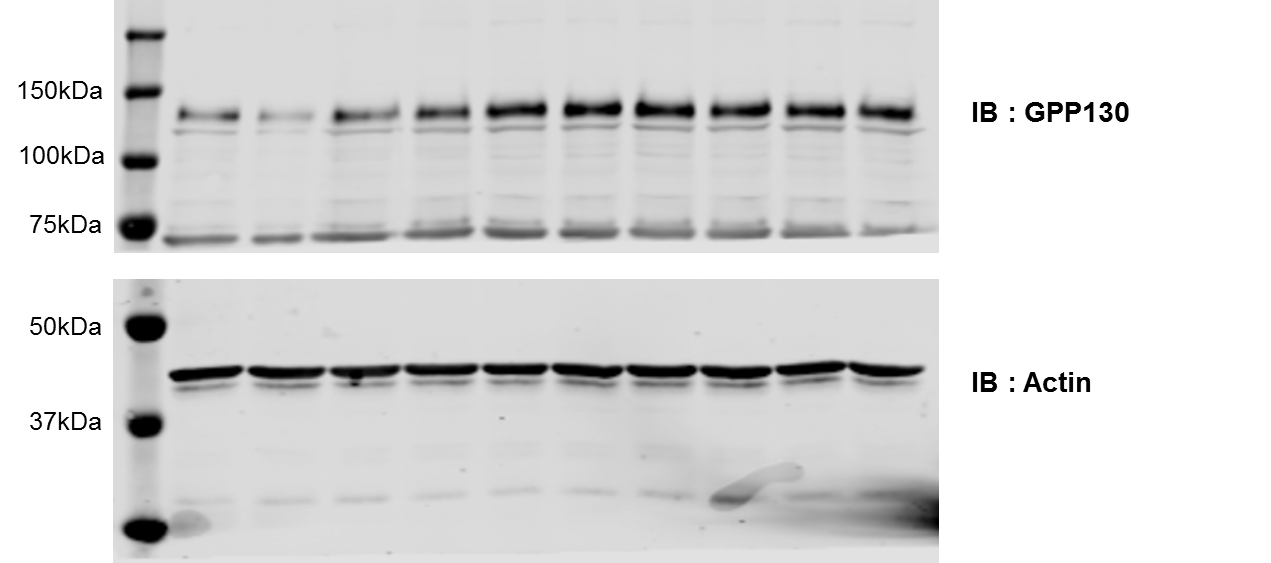
**

**Supplementary Figure 4. Full-length immunoblot images in Figure 3C.**

**
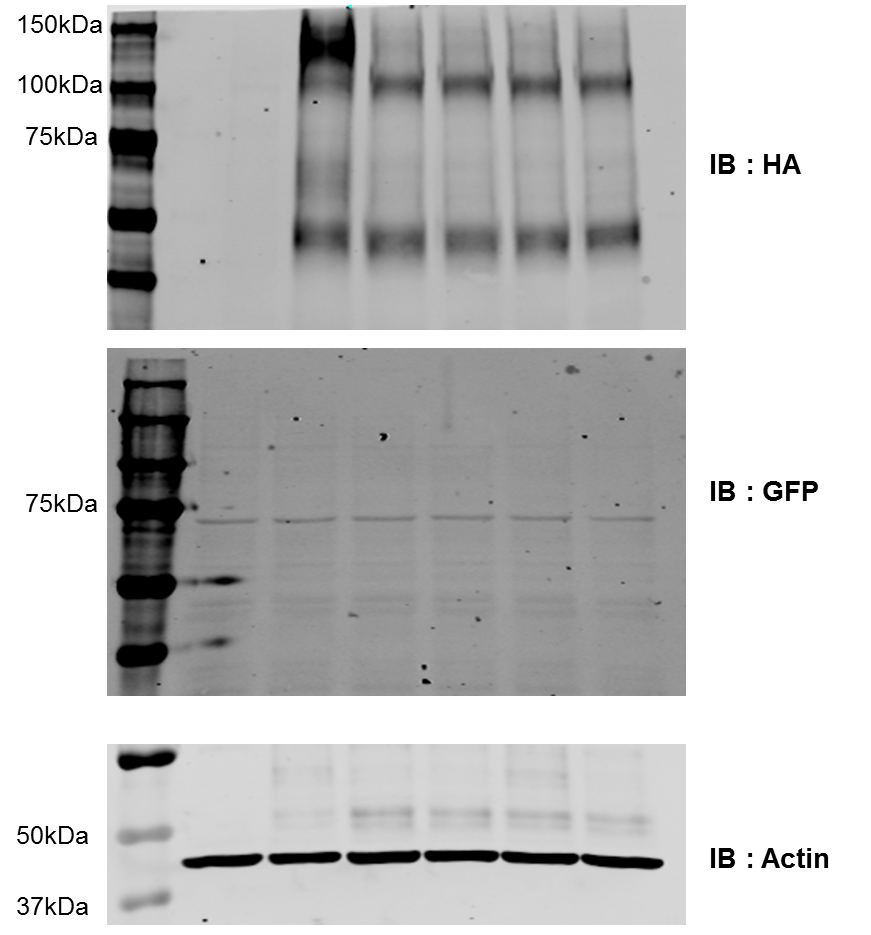
**

**Supplementary Figure 5. Full-length immunoblot images in Figure 3E.**

**
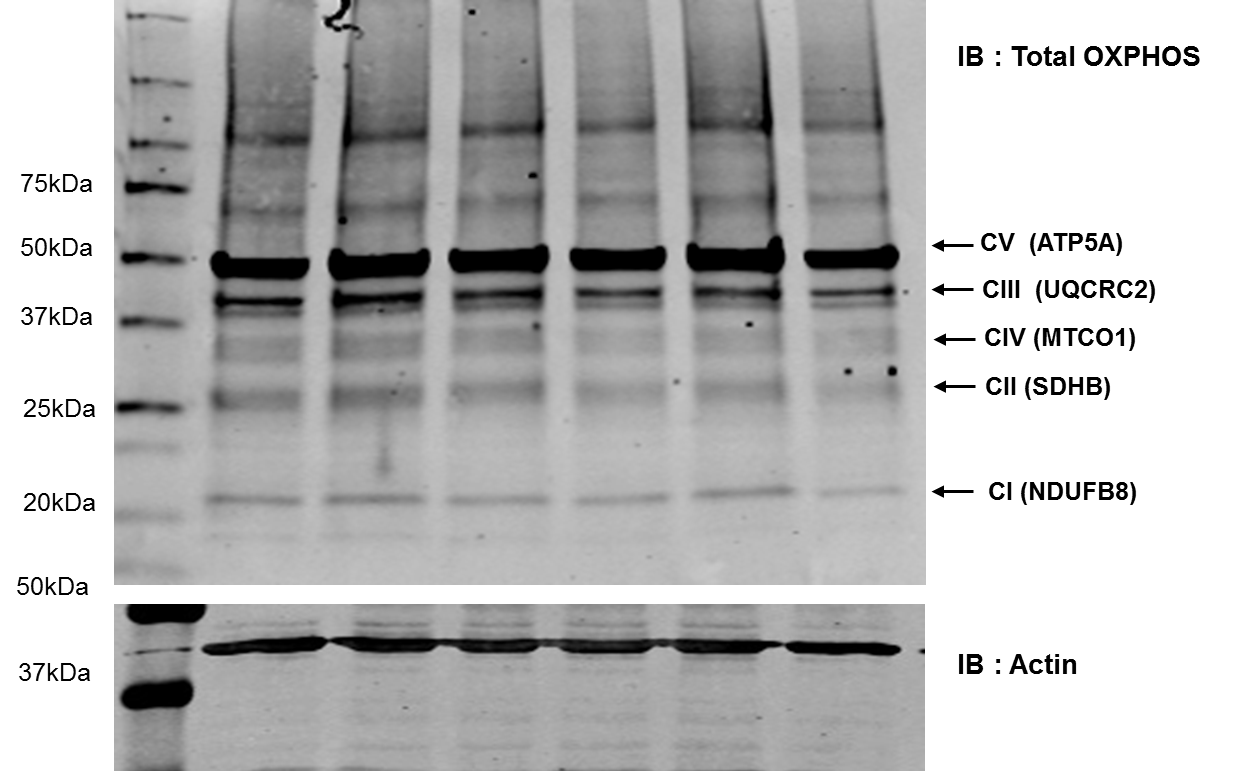
**

**Supplementary Figure 6. Full-length immunoblot images in Figure 6E.**


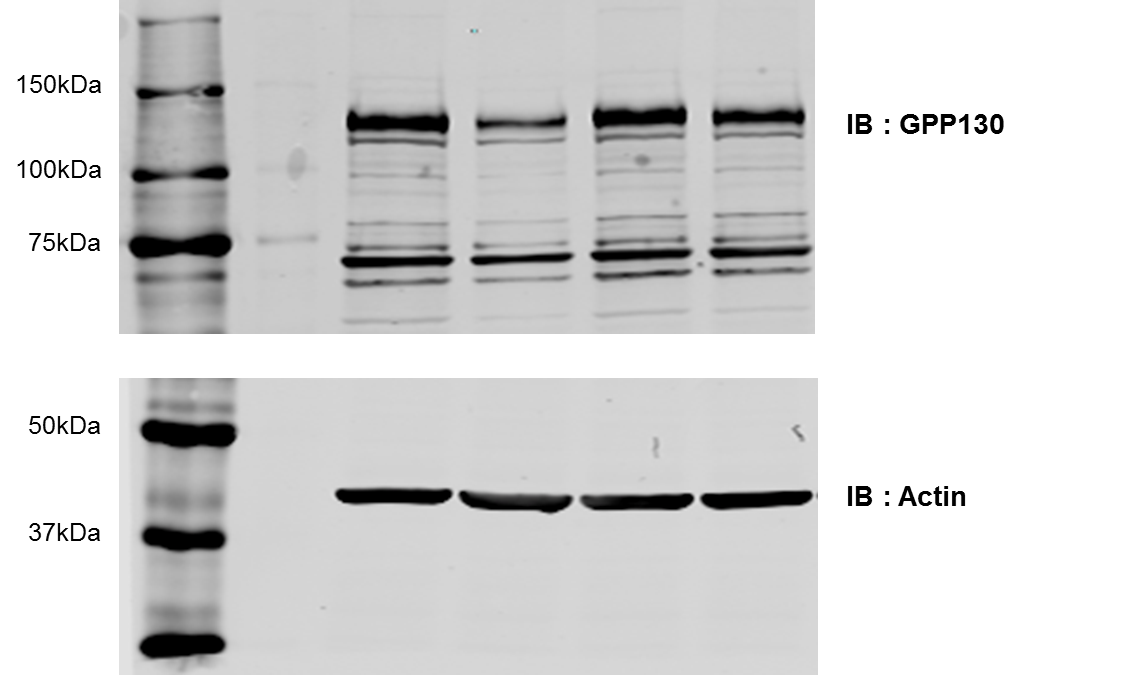


**Supplementary Figure 7. Full-length immunoblot images in Supplementary Figure 1.**
